# Model-based whole-brain effective connectivity to study distributed cognition in health and disease

**DOI:** 10.1162/netn_a_00117

**Published:** 2020-04-01

**Authors:** Matthieu Gilson, Gorka Zamora-López, Vicente Pallarés, Mohit H. Adhikari, Mario Senden, Adrià Tauste Campo, Dante Mantini, Maurizio Corbetta, Gustavo Deco, Andrea Insabato

**Affiliations:** Center for Brain and Cognition and Department of Information and Communication Technologies, Universitat Pompeu Fabra, Barcelona, Spain; Center for Brain and Cognition and Department of Information and Communication Technologies, Universitat Pompeu Fabra, Barcelona, Spain; Center for Brain and Cognition and Department of Information and Communication Technologies, Universitat Pompeu Fabra, Barcelona, Spain; Center for Brain and Cognition and Department of Information and Communication Technologies, Universitat Pompeu Fabra, Barcelona, Spain; Department of Cognitive Neuroscience, University of Maastricht, Maastricht, The Netherlands; BarcelonaBeta, Barcelona, Spain; Neuroplasticity and Motor Control Research Group, KU Leuven, Leuven, Belgium; Brain Imaging and Neural Dynamics Research Group, IRCCS San Camillo Hospital, Venice, Italy; Department of Neuroscience, Venetian Institute of Molecular Medicine (VIMM) and Padova Neuroscience Center (PNC), University of Padua, Italy; Department of Neurology, Radiology, and Neuroscience, Washington University School of Medicine, St. Louis, MO, USA; Center for Brain and Cognition and Department of Information and Communication Technologies, Universitat Pompeu Fabra, Barcelona, Spain; Institució Catalana de la Recerca i Estudis Avançats (ICREA), Barcelona, Spain; Institut de Neurosciences de la Timone, CNRS, Marseille, France

**Keywords:** fMRI, Cognition, Whole-brain dynamic model, Effective connectivity, Connectivity estimation, Machine learning, Classification, Biomarker, Network theory, Recurrent network, Dynamic communicability and flow, Community analysis

## Abstract

Neuroimaging techniques are now widely used to study human cognition. The functional associations between brain areas have become a standard proxy to describe how cognitive processes are distributed across the brain network. Among the many analysis tools available, dynamic models of brain activity have been developed to overcome the limitations of original connectivity measures such as functional connectivity. This goes in line with the many efforts devoted to the assessment of directional interactions between brain areas from the observed neuroimaging activity. This opinion article provides an overview of our model-based whole-brain effective connectivity to analyze fMRI data, while discussing the pros and cons of our approach with respect to other established approaches. Our framework relies on the multivariate Ornstein-Uhlenbeck (MOU) process and is thus referred to as MOU-EC. Once tuned, the model provides a directed connectivity estimate that reflects the dynamical state of BOLD activity, which can be used to explore cognition. We illustrate this approach using two applications on task-evoked fMRI data. First, as a connectivity measure, MOU-EC can be used to extract biomarkers for task-specific brain coordination, understood as the patterns of areas exchanging information. The multivariate nature of connectivity measures raises several challenges for whole-brain analysis, for which machine-learning tools present some advantages over statistical testing. Second, we show how to interpret changes in MOU-EC connections in a collective and model-based manner, bridging with network analysis. Our framework provides a comprehensive set of tools that open exciting perspectives to study distributed cognition, as well as neuropathologies.

## Author Summary

Brain connectivity measures have been increasingly used to study cognition and neuropathologies with neuroimaging data. Many methods have been developed with particular objectives, in particular predictability to obtain biomarkers (i.e., which brain regions exhibit changes in their interactions across conditions) and interpretability (relating the connectivity measures back to the biology). In this article we present our framework for whole-brain effective connectivity that aims to find the equilibrium between these two desired properties, relying on a dynamic model that can be fitted to fMRI time series. Meanwhile, we compare it with previous work. Concretely, we show how machine learning can be used to extract biomarkers and how network-oriented analysis can be used to interpret the model estimates in terms of brain dynamics.

## INTRODUCTION

The study of cognition has flourished in the recent decades because of the abundance of neuroimaging data that give access to brain activity in human subjects. Along the years, tools from various fields like machine learning and network theory have been brought to neuroimaging applications in order to analyze data. The corresponding tools have their own strengths, like predictability for machine learning. This article brings together recent studies based on the same whole-brain dynamic model in a unified pipeline, which is consistent from the model estimation to its analysis—in particular, the implications of the model assumptions can be evaluated at each step. This allows us to naturally combine concepts from several fields, in particular for predictability and interpretability of the data. We stress that our framework can be transposed to other dynamic models, while preserving the concepts underlying its design. In the following, we first review previous work on connectivity measures to set our formalism in context. After presenting the dynamic model (the multivariate Ornstein-Uhlenbeck process, or MOU), we discuss its optimization procedure to reproduce statistics of the fMRI/BOLD signals (spatiotemporal covariances), yielding a whole-brain effective connectivity estimate (MOU-EC). Then two MOU-EC-based applications are examined: machine learning to extract biomarkers and network analysis to interpret the estimated connectivity weights in a collective manner. Meanwhile, presenting details about our framework, we provide a critical comparison with previous studies to highlight similarities and differences. We illustrate MOU-EC capabilities in studying cognition in using a dataset where subjects were recorded in two conditions, watching a movie and a black screen (referred to as rest). We also note that the same tools can be used to examine cognitive alterations due to neuropathologies.

## CONNECTIVITY MEASURES FOR FMRI DATA

Among noninvasive techniques, functional magnetic resonance imaging (fMRI) has become a tool of choice to investigate how the brain activity is shaped when performing tasks (Cohen, 2018; Gonzalez-Castillo & Bandettini, 2017; Li, Wang, Yao, Hu, & Friston, 2012; Naselaris, Kay, Nishimoto, & Gallant, 2011). The blood-oxygen-level-dependent (BOLD) signals recorded in fMRI measure the energy consumption of brain cells, reflecting modulations in neural activity (Bartels, Logothetis, & Moutoussis, 2008; Becker, Reinacher, Freyer, Villringer, & Ritter, 2011; Ekstrom, 2010; Mitra et al., 2018). Since early fMRI analyses, a main focus has been on identifying with high spatial precision regions of interest (ROIs) in the brain that significantly activate or deactivate for specific tasks (Cordes et al., 2000; Laird et al., 2013; Walther et al., 2016). Because the measure of BOLD activity during task requires the quantification of a baseline, the brain activity for idle subjects became an object of study and developed as a proper line of research (Biswal, 2012; Snyder & Raichle, 2012). This revealed stereotypical patterns of correlated activity between brain regions, leading to the definition of the functional connectivity or FC (Buckner, 2010; Gillebert & Mantini, 2013). Together with studies of the anatomical connectivity using structural MRI (Hagmann et al., 2008; Sporns, Tononi, & Kötter, 2005), fMRI studies progressively shifted from focusing on specific ROIs to whole-brain analyses (Deco, Jirsa, & McIntosh, 2011). For example, high-order cognition involves changes in BOLD activity that are distributed across the whole brain (Chang, Gianaros, Manuck, Krishnan, & Wager, 2015; Rissman & Wagner, 2012), which cannot be restrained to a small set of preselected ROIs. The typical description of the whole-brain activity in these methods is a ROI-ROI matrix, which we refer to as connectivity measure.

### Glossary

•  Generative model: Model of (dynamic) equations that generates a signal to be fit to empirical data. This definition is different from the definition in statistics, where a generative model describes the joint probability distribution of observed and predicted variables (here the network activity), as opposed to a discriminative model that describes the conditional probability of the predicted variables with respect to the observed variables.
•  Linear Gaussian model (LGM): Generative model of Gaussian variables with linear relationships. Its output variables have a flat autocovariance (apart from zero time lag) and is used to model noisy data without temporal structure.
•  Multivariate autoregressive (MAR) process: Dynamic generative model in discrete time with linear relationships. Its output variables have both spatial and temporal correlations.
•  Multivariate Ornstein-Uhlenbeck (MOU) process: Dynamic generative model in continuous time with linear relationships (referred to as connections). It is the equivalent of the MAR process in continuous time.
•  Connections, interactions, and links: In the main text connections refer to a direct and causal relationship between nodes (ROIs) in a dynamic model, whereas interactions are used to describe network effects that may be mediated by indirect pathways in the network (for example with FC or dynamic flow). Links are used in the context of machine learning as a common term for EC connections or FC interactions.
•  Lyapunov optimization or natural gradient descent: Tuning procedure for the EC weights in the MOU network that fits the model FC covariance matrices to their empirical counterparts.
•  Classification pipeline: Succession of machine-learning algorithms that aims to learn the mapping from input vectors of “features” to output “labels” (or categories). Here we use neuroimaging connectivity measures to predict cognitive conditions (like the task performed by a subject).
•  Multinomial logistic regression (MLR): Linear multicategory classifier that assigns a coefficient to each input feature to predict the labels. It reduces to logistic regression for binary classification.
•  k-nearest neighbor (kNN): Nonlinear classifier that predicts the category of each new sample based on the most represented category over the k closest samples from the train set, given a distance metric or similarity measure.
•  Biomarker: Subset of observed features (here usually EC/FC links) that enable a robust classification, often paired with weights as in the case of a linear classifier.
•  Dynamic communicability: Measure of pairwise interactions between nodes (ROIs) in a network that takes indirect paths into account. In the present article, it corresponds to interactions over time for the MOU model.
•  Dynamic flow: Extension of dynamic communicability that incorporates the effect of input properties in the MOU model.

Recently, fMRI studies for both rest and tasks have also evolved to incorporate the temporal structure of BOLD signals in their analysis (Cabral et al., 2017; Ciuciu, Abry, & He, 2014; Gilson, Moreno-Bote, Ponce-Alvarez, Ritter, & Deco, 2016; Gonzalez-Castillo & Bandettini, 2017; B. J. He, 2011; Hutchison et al., 2013; Mitra, Snyder, Tagliazucchi, Laufs, & Raichle, 2015; Vidaurre et al., 2018), in addition to the spatial structure. Models have also been developed to formalize the link between the observed BOLD and the neuronal activity (Deco et al., 2013; Frässle et al., 2018; K. J. Friston, Harrison, & Penny, 2003; Gilson et al., 2016; Li et al., 2012; Messé, Rudrauf, Benali, & Marrelec, 2014; Vidaurre et al., 2018). This has led to many definitions for connectivity measures and variations thereof. In this introductory section we present fundamental concepts about connectivity measures that set our model-based approach in context (see also the Glossary for definitions).

### Model-Based Versus Model-Free Approaches

**Figure 1.**
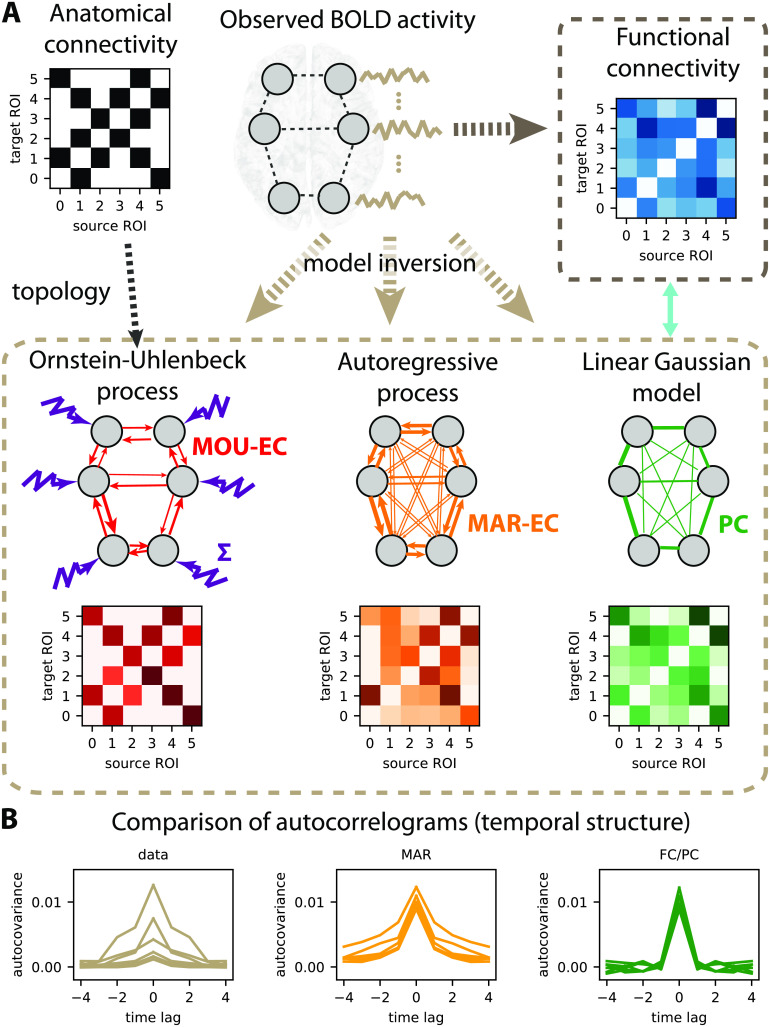
Connectivity measures. (A) Schematic diagram of six brain regions of interest (ROIs), whose fMRI activity is captured by several types of connectivity measures. Here the activity is generated using a directed matrix. The corresponding structural connectivity (SC, black matrix) indicates the topology of anatomical connections between the six ROIs, which can be estimated using structural MRI. Functional connectivity (FC, blue matrix), here calculated using the Pearson correlation of the BOLD signals. The linear Gaussian model (LGM) assumes activity and corresponds to partial correlations (PC, green matrix). The multivariate autoregressive (MAR, orange matrix) assumes linear dynamics while taking the temporal structure of the data into account. Effective connectivity (EC, red matrix), which is the focus of the present article, depends on the choice for dynamic model, as well as input properties. Here the dark brown dashed box groups together the connectivity measures that involve a model inversion for their estimation, as compared with the light brown box that can be directly computed from the observed data. (B) Autocorrelograms of the data (in light brown, left plot) for six ROIs and two network models (middle and right plots). The linear Gaussian model related to the FC and PC (in green) has a flat autocorrelogram away from the central peak. In contrast, the profile of the MAR process (in orange) has a decaying exponential profile.

A recurrent discussion opposes model-free and model-based approaches in neuroscience, which also applies for connectivity measures. For instance, FC calculated using Pearson correlation between time series (blue matrix in Figure 1A) can be directly calculated from data—see Equation B.2 in the Supporting Information—and may thus be seen as model-free. However, the interpretation of the FC values for the pairs of ROIs in the matrix is based on the theory of linear Gaussian models (LGMs), which assumes multivariate normal distributions for the data. The Pearson correlation is a connectivity measure that can be directly calculated on the generated activity. The underlying connectivity in the LGM, called the precision matrix, is closely related to the partial correlations (PC) represented by the green matrix in Figure 1A. The PC symmetric matrix describes the undirected linear relationships between Gaussian variables that result in the observed correlations. Our viewpoint is thus that connectivity measures are always related to a model, so it is crucial to bear in mind their assumptions when interpreting their values. Note that other classifications of methods have been proposed and discussed in previous work, like directed functional connectivity measures versus effective connectivity measures (K. Friston, Moran, & Seth, 2013), state-space models versus structural models for BOLD dynamics (Valdes-Sosa, Roebroeck, Daunizeau, & Friston, 2011) and network-wise methods versus pairwise inference methods (Bielczyk et al., 2019).

In the literature of whole-brain modeling, three families of connectivity measures have emerged:

•  Structural connectivity (SC): It measures the density or probability of anatomical pathways that connect two ROIs, mostly via the white matter (Schmahmann & Pandya, 2007). This led to the definition of the human connectome at the whole-brain level (Hagmann et al., 2008; Sporns et al., 2005).
•  Functional connectivity (FC): Pairwise statistical dependencies between the observed activity of ROIs (Buckner, 2010; Gillebert & Mantini, 2013). Apart from the Pearson correlation of the BOLD signals (Biswal, 2012; Snyder & Raichle, 2012), other common FC measures include mutual information (Hlinka, Palus, Vejmelka, Mantini, & Corbetta, 2011) and synchrony of instantaneous phases (Cabral, Hugues, Sporns, & Deco, 2011; Cabral et al., 2017). Conceptually, FC corresponds to a measure that can be applied to multiple time series, either the data or the model activity.
•  Effective connectivity (EC): In this article we define EC as a measure of the directional relationships in a dynamic model. The original concept arises from electrophysiology (Aertsen, Gerstein, Habib, & Palm, 1989), where EC determines how the stimulation of a neuron affects a target neuron (e.g., combining the source-to-target synaptic weight and the excitability of the target). It was then brought to neuroimaging in the 2000s when building models of the BOLD response and further developed in the context of the dynamic causal model (DCM), usually interpreted as neuronal coupling (K. Friston, 2011; K. J. Friston et al., 2003; Valdes-Sosa et al., 2011). Note that it is also still used close to its original formulation when measuring stimulation-driven responses in neuroimaging (Lafleur, Tremblay, Whittingstall, & Lepage, 2016).

To go beyond statistical relationships, the combination of several types of data requires a model, as with the SC and FC that are combined in the EC model (see Figure 1A). Therefore the choice of the model ingredients, especially their dynamics, has important implications that we detail in the following.

### Choice of Model and Interpretability

In our framework we borrow the EC terminology that can be understood in a broad sense as the directed connectivity in a generative model, here for BOLD activity. When using the LGM related to Pearson correlations taken as FC, one can take the partial correlation (PC, green matrix in Figure 1A) as “LGM-EC.” As illustrated by the flat green autocorrelogram for nonzero time lags in Figure 1B, the generated signals by the linear Gaussian model are independently and identically distributed (i.i.d.) variables and do not have temporal correlations. In contrast, the multivariate autoregressive process (MAR, in orange in Figure 1A–B) has a directed connectivity (asymmetric matrix) and produces temporally correlated signals. When these models are fitted to data, they do not capture the same part of the data structure. When the MAR model is considered, the estimation results in directed connectivity that depends on the spatiotemporal correlation structure of the observed activity (or spatiotemporal FC). However, the linear Gaussian model does not “naturally” give directed connectivity when fitted to the spatial FC. Note that techniques based on optimization with regularization have been developed to enforce directionality in the estimated connectivity (Schiefer et al., 2018), though.

EC usually describes causal and directional relationships that interplay with other dynamic parameters in the model to determine the global network pattern of activity. When optimizing a model to reproduce BOLD signals, the parameter can be seen as representations or “projections” of the BOLD signals, in a top-down or data-driven approach (Valdes-Sosa et al., 2011). Importantly, the estimated EC depends on the choice for the model dynamics. For example, the DCM was developed to formalize the link between neural and BOLD activities by explicitly modeling the hemodynamics (K. Friston, 2011; K. J. Friston et al., 2003; K. Stephan, Harrison, Penny, & Friston, 2004). The DCM-EC is thus the directed connectivity between the ROIs and determines their activities, from which the BOLD signals are generated via a hemodynamic response function (HRF).

We keep in mind that all interpretations of fMRI signals sit on the hypothesis that correlated BOLD activity between brain areas reflects the activity of their neuronal populations (David et al., 2008; Goense & Logothetis, 2008; Y. He et al., 2018; Matsui, Murakami, & Ohki, 2018), which in turn mediates the transmission of neuronal information (Fries, 2005). However, many metabolic mechanisms like breathing (which are usually ignored in whole-brain models) alter the BOLD signals (Power et al., 2018), and the adequate procedure to minimize the corruption of data and obtain a satisfactory signal-to-noise ratio is still under debate (Parkes, Fulcher, Yücel, & Fornito, 2018).

A distinct line of research (Cabral et al., 2011; Deco et al., 2013; Proix et al., 2016; Sanz-Leon, Knock, Spiegler, & Jirsa, 2015) focuses on the development of whole-brain models in a more bottom-up fashion (model-driven), combining various datasets and biologically inspired mechanisms, such as oscillators to produce rhythms observed in the brain. Such approaches allow for the study of the influence of specific parameters (as ingredients in the model) in collectively shaping the global network activity. Compared with connectivity estimation, a major distinction of many of those models is that SC is taken as the intracortical connectivity to focus on the influence of the local dynamics in shaping network activity patterns; see Messé et al. (2014) for a review.

### Consistency of Pipeline From Preprocessed Data to Modeling and Analysis

The incentive for novelty has fostered the development of many methods in computational neuroscience. This makes results difficult to compare, especially for resting-state fMRI studies where no ground truth is available. Another caveat concerns network analysis when it is applied to connectivity estimates from time-varying signals (e.g., BOLD) with metrics (e.g., to detect communities) that do not genuinely relate to the physical values of the data. Our practical answer to this point is thus to develop for the same dynamic model a set of analysis tools, covering parameter inference (Gilson et al., 2016) to machine learning and network analysis. Although the mathematical tractability of the model limits the richness of its dynamic repertoire compared with more elaborate bottom-up models (Deco et al., 2013; Sanz-Leon et al., 2015), it provides an intuitive understanding of the roles of parameters as well as analytical derivations for the parameter estimation or model interpretation. We believe that such an effort for formalization and consistency is beneficial to the field, both for didactic purpose and for designing tailored methodology for complex models.

**Figure 2.**
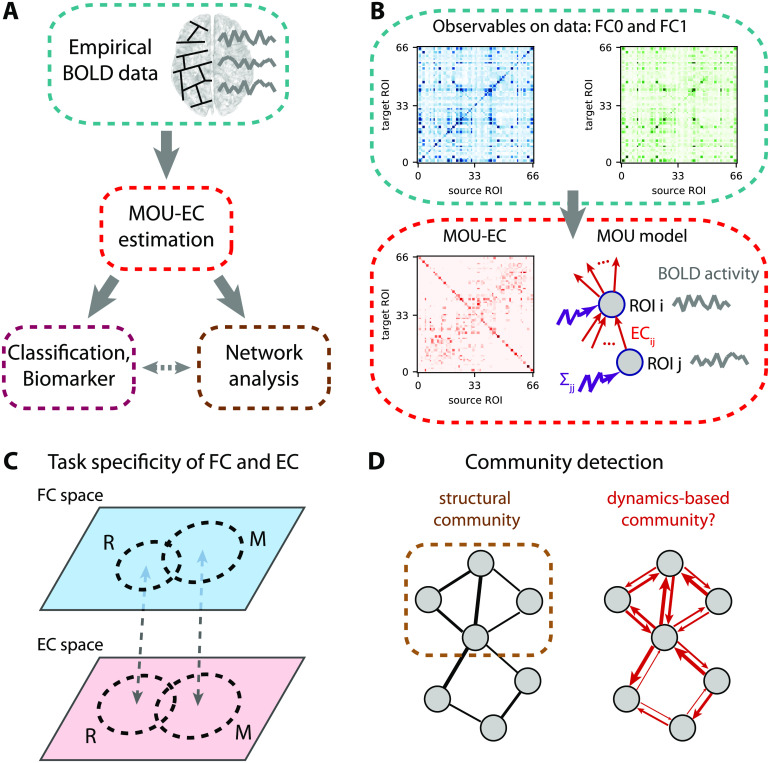
EC-based analysis of empirical BOLD signals. (A) Pipeline for brain coordination analysis using MOU-EC. For each fMRI session, the model is optimized by tuning its parameters, especially its connectivity, to reproduce the statistics of the BOLD signals (depicted in the right side of the brain). A schematic parcellation is represented on the left hemisphere of brain. The estimated MOU-EC can then be used to predict cognitive states using classification algorithms. On the other hand, MOU-EC can be analyzed as a graph (or network) to uncover collective properties. A contribution of our approach is to link the network-oriented analysis with machine-learning techniques. (B) From the BOLD signals, two FC matrices are calculated, FC0 without time lag in blue and FC1 with a time lag of 1 TR in green. The model parameters (MOU-EC and Σ) are optimized to reproduce these empirical FC matrices. Classification focuses on the model connectivity (e.g., MOU-EC), but the influence of Σ will be incorporated in the network analysis. See Figure S1 for further details. (C) Schematic representation of the FC and MOU-EC matrices (one sample per session) in their own spaces for two cognitive states (R for rest and M for movie). For each connectivity measure, the prediction of R versus M is robust when the sample distributions represented by the dashed circles do not overlap. (D) The left diagram represents community detection in a structural undirected network. The right diagram represents the MOU-EC networks, from which we want to define dynamics-based communities.

The present article follows the pipeline displayed in Figure 2A. The second section presents the dynamic model and our recent optimization procedure (Gilson, Deco, et al., 2018; Gilson et al., 2016) to estimate MOU-EC for whole-brain fMRI data (Figure 2B). It also discusses technical aspects in relation with other models used with neuroimaging data. The third section shows how connectivity measures can be used to predict cognitive states (Figure 2C) and uncover their underlying structure. Importantly, the use of datasets with multiple tasks allows for an in-depth benchmarking of the connectivity measures. It also highlights some advantages of machine learning over statistical testing in making use of the multivariate nature of connectivity measures, transposing and abstracting concepts presented in a recent study for subject identification (Pallarés et al., 2018). The fourth section bridges with network analysis (Gilson et al., 2019; Gilson, Kouvaris, Deco, & Zamora-López, 2018), interpreting changes in MOU-EC connections in a collective and model-based manner. In particular, it adapts community detection to our dynamic model (Figure 2D).

## CAPTURING THE WHOLE-BRAIN BOLD DYNAMICS WITH MOU-EC

Using a generative model to reproduce the BOLD statistics, the optimized MOU-EC can be seen as an estimate that extracts spatiotemporal information about the BOLD *dynamics*. In fact, the main innovation of the modeling is the optimization procedure in Figure 2B (Gilson et al., 2016). This section reviews important points about the underlying multivariate Ornstein-Uhlenbeck (MOU) dynamic model and its tuning. Further mathematical details can be found in the Supporting Information (see Figure S1). Our approach aims to combine several key aspects:

•  **Whole-brain** connectivity estimates are necessary to properly take into account the experimentally observed distributed information across distant regions (Chang et al., 2015; Deco et al., 2011), without a priori selection of ROIs.
•  From the overall **spatiotemporal structure** of BOLD signals, the dynamic model only reproduces their covariances (without and with time lag, see Figure 2B). This concerns the same fast timescale as recent studies (Mitra, Snyder, Hacker, & Raichle, 2014; Mitra et al., 2015), which also demonstrated the influence of behavioral states such as sleep versus wake. The choice of BOLD covariances to be reproduced by the model is supported by previous results that showed that most of the information was in the second-order statistics of BOLD signals (Hlinka et al., 2011).
•  **Causality** is inherent to the concept of EC (K. Friston, 2011; K. J. Friston et al., 2003; Valdes-Sosa et al., 2011) that is represented by the directed connections in the dynamic model (red arrows in Figure 2B). From the estimated MOU-EC that best reproduces the data, we interpret strong MOU-EC weights as causal relationships and can examine their asymmetry to evaluate for a pair of ROIs which one drives the other. The optimization can deal with common inputs for ROIs, to explain the observed correlated activity by an interplay between the directed connectivity and correlated inputs (Gilson, Deco, et al., 2018).
•  The MOU-EC **topology** is the interregional infrastructure, namely which connections exist and which do not in the brain network. When SC is available, its binarization (black matrix in Figure 1A) can be used to constrain the MOU-EC topology in order to reduce the number of parameters to estimate. This enforces the model to “explain” changes in the FC by existing connections only, in contrast to PC and MAR (Figure 1A). It is thus important to remember that MOU-EC estimates are not related to SC values.
•  The optimization procedure tunes all MOU-EC weights while taking into account network effects. For each fMRI session, we obtain a **multivariate** estimate of more than 1,000 parameters that represent the dynamical “state” of the brain activity. This contrasts with previous models that used the symmetric SC as connectivity matrix in dynamic models and focused on choosing or tuning the nodal dynamics with a few parameters only (Deco et al., 2013; Messé et al., 2014; Sanz-Leon et al., 2015).
•  A limitation of the model estimation procedure up to now is the assumption of stationarity for the BOLD signals over each fMRI session, which limits our approach to ongoing nonswitching tasks.
•  Another limitation of MOU-EC for the interpretation in terms of neuronal coupling is the absence of explicit hemodynamics in the model. This choice comes from the priority given so far to the estimation robustness (with simpler dynamics) over the biological interpretability, as will be discussed later.

### Multivariate Ornstein-Uhlenbeck Dynamics as Generative Model for BOLD Signals

Formally, our model-based analysis is based on the multivariate Ornstein-Uhlenbeck process that is described by Equation C.1 in the Supporting Information. It corresponds to a network with linear feedback that is the equivalent in continuous time of the discrete-time multivariate autoregressive (MAR) process. These dynamic systems with linear feedback have been widely used in neuroscience to model the propagation of fluctuating activity, mixing “noise” and structure, for example in modeling single neuronal dynamics (Burkitt, 2006), relating the connectivity and activity in a network (Galán, 2008) and defining neural complexity (Barnett, Buckley, & Bullock, 2009; Tononi, Sporns, & Edelman, 1994). It also corresponds to the linearization of nonlinear population models like Wilson-Cowan neurons (Wilson & Cowan, 1973).

The choice for the MOU dynamics is motivated by the balance between simplicity, which ensures tractable analytical calculation, and richness of the generated activity when modulating the parameters (especially the MOU-EC weights). Thus, it is well adapted to whole-brain data with parcellation involving many ROIs (≥ 100). In addition, the MOU dynamics implies exponential decaying autocovariances in the model, which have similar profiles to the empirical data (see the left plot in Figure 1B corresponding to straight lines in log-plot in Figure S1).

Intuitively, it can be understood as a network when fluctuating activity (akin to noise) is generated at each ROI and propagates via the recurrent EC. In other words, MOU-EC (red matrix in the bottom box) acts as a “transition matrix” and quantifies the propagation of fluctuating BOLD activity across ROIs. The MOU-EC matrix is usually sparse when its topology is constrained by SC (black matrix) to match anatomical white matter connections between ROIs. The fluctuating activity for all ROIs is described by their (co)variance matrix Σ, which is diagonal in the present case (see the purple vector of variances). In a previous study, cross-correlations for Σ also involve common inputs to homotopic sensory ROIs (Gilson, Deco, et al., 2018).

### Parameter Estimation Capturing Network Effects in BOLD Propagation

To capture the BOLD dynamics (i.e., propagation of fluctuating activity), we use the two BOLD covariance matrices FC0 and FC1 in Figure 2B, without and with time lag, respectively. This also ensures a one-to-one mapping between an FC configuration (a pair FC0 and FC1) and a MOU-EC configuration. This choice is in line with previous adjustments of DCM to model the resting state that relied on the cross-spectrum, namely the Fourier transform of covariances with all possible time lags (K. Friston, 2011). It is richer than fitting only an FC matrix without time lag (Deco et al., 2013; Messé et al., 2014) and complies with a recent study of the task-dependent modification of the BOLD temporal structure at short timescales (Mitra et al., 2015).

The MOU-EC estimate is obtained for the minimum model error in reproducing the empirical FC0 and FC1 for each session (see the fit plot between the boxes in Figure S1). The optimization is akin to a “natural” gradient descent (Amari, 1998) in that it takes into account the nonlinearity of the mapping from MOU-EC to the covariances FC0 and FC1. This arises because of the network feedback (even though linear) and may result in strong correlations (in FC) for disconnected ROIs (EC = 0) provided strong indirect pathways connect them (via other ROIs). Appendix D in the Supporting Information provides the mathematical details of the optimization, which applies the gradient descent to both MOU-EC and Σ (Gilson, Deco, et al., 2018), extending the initial formulation with a heuristic optimization for Σ (Gilson et al., 2016).

For illustration purpose, this article uses a whole-brain parcellation consisting with 66 ROIs. The SC density is 28%, giving 1,180 MOU-EC weights to estimate (see Appendix A in the Supporting Information). Each fMRI session has 300 time points separated by TR = 2s, so the number of data points is 66 × 300 ≃ 2.10^4^, about 16 times larger than the number of model parameters. EC should be like a summary of the BOLD signals: informative (not too short), but extracting and compressing relevant information (not too long). Our method was also successfully applied to the AAL (Automated Anatomical Labeling) parcellation with 116 ROIs and sessions of 5 min (Pallarés et al., 2018). Typically, the MOU-EC estimation for a session and about 100 ROIs takes less than a minute of computation time on a desktop computer. For more refined parcellation or shorter sessions, the FC matrices may become quasisingular and the model estimates are expected to be noisier. The reduction of the number of parameters to estimate by using SC is crucial to work at the level of individual fMRI session and avoid overfitting. Here, overfitting would correspond to the situation where many distinct MOU-EC configurations in the model give very similar pairs of FC0 and FC1 matrices, giving a larger overlap for MOU-EC than in Figure 2C and the same small overlap for FC. The comparison between FC and MOU-EC as multivariate representation of cognitive states will be the focus of the next section. An important conceptual difference between MOU-EC and SC is that MOU-EC accounts for the dynamical properties of the brain activity, which are modulated when engaging a task. In other words, MOU-EC is hypothesized to account for the concentration of receptors or neurotransmitters, local excitability, and so on not only the density of the synaptic fibers.

### Comparison With Other Approaches to Extract Information From BOLD Signals

Other approaches have been proposed to characterize activity “states” based on the temporal structure of BOLD signals. For example, the “dynamic FC” relies on sliding windows of several tens of TRs (Gonzalez-Castillo & Bandettini, 2017; Hutchison et al., 2013; Park & Friston, 2013; Preti, Bolton, & Van De Ville, 2017), thereby focusing on changes in correlation patterns over minutes. Shorter timescales have been explored using instantaneous phases obtained using the Hilbert transform on the BOLD signals (Cabral et al., 2017) or hidden Markov models (HMMs; Bolton, Tarun, Sterpenich, Schwartz, & Van De Ville, 2018; Vidaurre et al., 2018). In contrast, the MOU-EC describes the BOLD propagation averaged over a session while assuming stationarity, as calculated in the corresponding statistics (covariances without lag and with a lag of 1 TR). Note that the “transition matrix” analogy for EC is at the level of the BOLD activity, not of hidden states as in the case of HMMs. Moreover, it does not involve a dynamic modulation of EC as used in the DCM (K. Friston, 2011; Li et al., 2012; Park & Friston, 2013).

A key innovation to tackle whole-brain fMRI data is the optimization constrained by SC that determines the network topology. This means the model has to explain the spatiotemporal FC structure using the existing connections only. The “prior” information related to SC avoids the preselection of ROIs and can be seen as an alternative to model comparison in choosing the best topology using structural equations (James et al., 2009; McIntosh & Gonzalez-Lima, 1994), Granger causality analysis (Goebel, Roebroeck, Kim, & Formisano, 2003), and early versions of the DCM (K. J. Friston et al., 2003; Valdes-Sosa et al., 2011)—note that a recent DCM study incorporates SC for the network topology (Sokolov et al., 2018). Likewise, the MOU-EC density usually depends on the threshold applied to SC and the best choice can be decided using model comparison, although the formulation may not be as natural as in the Bayesian framework.

Several fundamental properties were discussed a few years ago about the defining concepts of EC and DCM (Valdes-Sosa et al., 2011). Beyond technical details, three main points are that DCM-EC corresponds to the connectivity weights in a dynamic model, that the model incorporates the hemodynamic response function, and that the estimation captures the BOLD dynamics, including the subsampling related to the low time resolution of BOLD signals. The EC terminology was borrowed from the DCM literature (K. Friston, 2011) because of the model-based aspect. The MOU-EC estimation was developed to solve the trade-off between robust estimation and application to large brain network (70+ ROIs) by using linear dynamics (Gilson et al., 2016). Since then, the DCM has been applied to whole-brain fMRI data (Frässle et al., 2018; Razi et al., 2017).

The FC0 and FC1 matrices in Figure 2B embody the spatiotemporal BOLD structure in the range of “high” frequencies close to the Nyquist frequency equal to 0.5 Hz. The recent extension of the DCM to analyze resting-state fMRI data reproduces the BOLD statistics via the cross-spectrum (Frässle et al., 2018; K. J. Friston, Kahan, Biswal, & Razi, 2014; Razi et al., 2017), which is in line with our approach. Recall that this contrasts with earlier versions of the DCM that reproduced the BOLD time series themselves for stimulation protocols (K. J. Friston et al., 2003; Li et al., 2012). Because it works in continuous time, the MOU model deals with the BOLD subsampling (Gilson et al., 2016), unlike estimation methods relying on the discrete-time multivariate autoregressive process that may be sensitive to the subsampling of the BOLD signals (Seth, Chorley, & Barnett, 2013). Moreover, the observed lags between ROIs in FC—similar to cross-correlograms (Mitra et al., 2014, 2015)—are explained by the combination between the estimated inputs Σ and MOU-EC (Gilson, 2018).

The time constant *τ*_*x*_ in the MOU model is identical for all ROIs in Equation C.1 in the Supporting Information, which corresponds to an abstraction of the HRF waveform response that was reported with a decay of 5 to 10 s (d’Avossa, Shulman, & Corbetta, 2003). An important difference of our model compared with the underlying dynamic model behind DCM (K. J. Friston et al., 2003; Valdes-Sosa et al., 2011), as well as other bio-inspired network models (Deco et al., 2013), is the absence of an explicit HRF to link the neural activity to the measured BOLD signals (Boynton, Engel, Glover, & Heeger, 1996; K. Friston, Mechelli, Turner, & Price, 2000; K. Stephan et al., 2004). Note that applications of the DCM to resting-state data involve a linearization of the HRF (Frässle et al., 2018; Razi et al., 2017). Therefore, a foreseen extension of our approach is that of a state-space model with the MOU process generating the neuronal activity that is convolved with a linear HRF filtering (Sauvage, Hubert, Touboul, & Ribot, 2017). The challenge is to keep the gradient-descent optimization tractable, as was previously done with Granger causality analysis (Barnett & Seth, 2015; Faes, Stramaglia, & Marinazzo, 2017). Incorporating the HRF may improve the reliability of the estimated EC (Gitelman, Penny, Ashburner, & Friston, 2003; Olszowy, Aston, Rua, & Williams, 2019). Note that an alternative consists in performing the deconvolution of the BOLD signals with respect to a HRF before analyzing the obtained neuronal signals, as was done with Granger causality analysis (David et al., 2008; Goodyear et al., 2016; Sathian, Deshpande, & Stilla, 2013; Wheelock et al., 2014) or other connectivity analysis (Ryali, Supekar, Chen, & Menon, 2011).

## MACHINE LEARNING FOR EXTRACTING MULTIVARIATE BIOMARKER SPECIFIC TO COGNITIVE STATES

Beyond their goodness of fit and how many “realistic” biological mechanism they might incorporate, models can be used to extract information from data in a top-down approach. Once tuned, the model parameters can indeed be used to discriminate cognitive conditions, as a representation of the observed data (BOLD signals here). This conceptually differs from finding the generative model of activity with the best fit. Nonetheless, it is expected that a model with poor fit hardly extracts any relevant information. Many recent studies have used connectivity measures to predict which tasks are performed by the subjects in the scanner (Gonzalez-Castillo et al., 2015), the pathological conditions of patients (Kurth, Moyse, Bahri, Salmon, & Bastin, 2015; Rahim, Thirion, Bzdok, Buvat, & Varoquaux, 2017), and individual identity (Amico & Goñi, 2018; Calhoun, Lawrie, Mourao-Miranda, & Stephan, 2017; Finn et al., 2015; Miranda-Dominguez et al., 2014). Machine learning is the standard for identifying biomarkers of neuropathologies (Varoquaux et al., 2017) and is also widely used in voxel-based analysis for cognitive tasks (Naselaris et al., 2011; Walther et al., 2016). However, it is less frequently applied for cognition studies using connectivity measures (Varoquaux & Poldrack, 2019). For a given connectivity measure, a biomarker is a subset of connections (or links) that enables the robust identification of a category of fMRI sessions, such as a weighted sum of the matrix elements that exceeds a threshold for the multinational logistic regression (MLR). In practice, changes in BOLD activity across cognitive conditions are mixed with individual traits and session-to-session variability. Disentangling these contributions is the key to obtain efficient biomarkers (Pallarés et al., 2018).

The present section addresses the following questions:

•  How to cope with the multivariate nature of connectivity measures (e.g., > 1,000 MOU-EC links)? We illustrate some advantages of machine learning compared with statistical testing, about multiple comparisons and beyond.
•  What does classification tell about the underlying model? Linear and nonlinear classifiers apply different metrics on the estimated parameters, revealing the distribution of task-specific changes across brain regions.
•  In a complex environment (e.g., many tasks), can we uncover a hierarchy in cognitive states (subgroups of tasks)?

**Figure 3.**
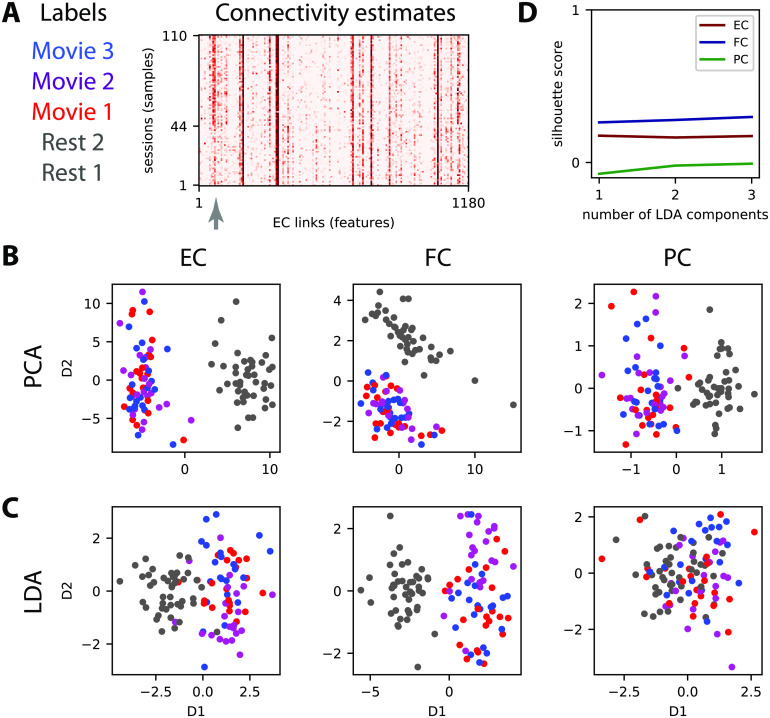
Dimensionality reduction of the connectivity measures. (A) Connectivity estimates (here vectorized EC) for five sessions, two for rest and three for movie, for each of the 22 subjects. Each of the three movie sessions corresponds the three movie sessions each of corresponds to a distinct part (10 min) of a movie watched and listened to by the subjects. The gray arrow indicates connections (or links) that strongly differ between rest and movie. (B) Principal component analysis (PCA) applied to the vectorized EC, FC, and PC (one for each of the 110 sessions) where the same colors as in panel A correspond to the 4 tasks. For PCA the task labels are not used in the dimensionality reduction (unsupervised algorithm). (C) Similar plot to panel B with linear discriminant analysis (LDA) for which the labels are used (supervised learning) to separate the four tasks for the 110 sessions. (D) The silhouette coefficient measures the quality of the clustering for the four dot clouds when varying the number of LDA components (see x-axis).

Adopting the language from machine learning, “samples” refer to fMRI sessions and the links of the connectivity measures are “features” whose values are used to discriminate “labels”, which are the types of fMRI sessions here. To illustrate our framework, we use data that were previously analyzed (Demirtaş et al., 2019; Gilson, Deco, et al., 2018; Hlinka et al., 2011; Mantini et al., 2012), whose details are summarized in Appendix A in the Supporting Information. Subjects in the scanner were recorded fixating on a point in a black screen (two rest sessions R1 and R2) or a movie. The entire movie session was divided in three sessions (M1, M2, and M3), each corresponding to a different part of the movie. The vectorized MOU-EC estimates are displayed for all subjects and sessions in Figure 3A. The question is then whether connectivity measures can capture information to identify the movie sessions individually. For example, the MOU-EC links indicated by the gray arrow exhibit strong changes between rest and movie (with small and large values, respectively).

### Variability of Connectivity Measures With Respect to Cognitive Labels

Before classification, the data structure can be explored to evaluate the similarity or distance across conditions between the connectivity measures, as representations of the BOLD signals. For this type of (passive) task, BOLD synchronization patterns during movie viewing between brain areas (e.g., visual and auditory) as reflected by intersubject correlations (Hasson, Nir, Levy, Fuhrmann, & Malach, 2004) are expected to be also captured by the connectivity measures (Gilson, Deco, et al., 2018). The gray arrow in Figure 3A indicates some MOU-EC links with strong changes between rest and movie.

A usual technique for multivariate data is dimensionality reduction, for example using principal component analysis (PCA; Gonzalez-Castillo et al., 2015) and independent component analysis (ICA; V. D. Calhoun, Liu, & Adali, 2009). Here we compare two linear transformations, the unsupervised PCA in Figure 3B with the supervised linear discriminant analysis (LDA) in Figure 3C. The principal component is the direction in the high-dimension space with largest variability across the samples, and successive components are ranked in decreasing order for their corresponding variability. It is called unsupervised because it does not use the task labels to compute the components, as can be seen for FC where the first component is not related to the task labels. In comparison, the components for LDA are ranked according to their variability *with respect to task labels*. This can be seen for FC where the first component for PCA does not reflect the separation between movie and rest (the second component does).

The separation of the tasks viewed using the connectivity measures can be evaluated using silhouette coefficients that measure the clustering quality (Rousseeuw, 1987), ranging from 1 for separated dot clouds to −1 for completely overlapping dot clouds. Applied to the LDA coordinates in Figure 3D, we see a slight increase in silhouette coefficient when incorporating more components to separate the four clouds. This reflects the difficulty of the four-task discrimination, as compared with that for the two tasks where a single component is sufficient (Demirtaş et al., 2019). The viewpoint here is that of clustering, assuming that the reduced space allows for isolating the clouds to characterize the cognitive states (Gonzalez-Castillo et al., 2015). In the following, we rely on various classification techniques for the task discrimination.

### Cross-Validation for Assessing the Generalization Capability of Connectivity-Based Classification

**Figure 4.**
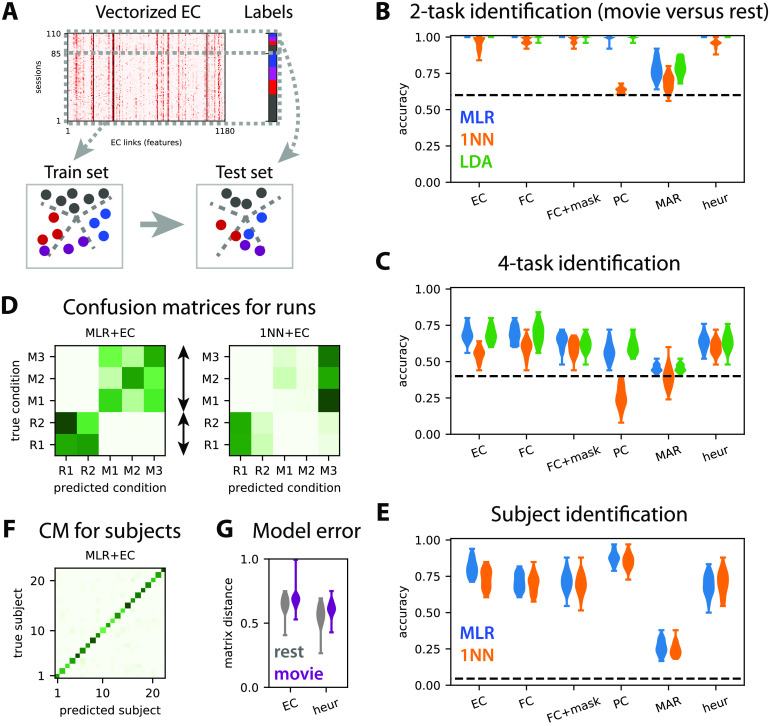
Train-test classification for task or subject identification. (A) The train-test procedure consists in splitting the sessions in a train set and a test set. The train set is used to optimize the classifier (here find the best boundaries between labels; see dashed gray lines), whose accuracy is then measured on the test set. (B) Accuracy for the classification of test sessions to discriminate movie versus rest (two labels) for the MLR, 1NN, and LDA classifiers, as indicated by the colors. The connectivity measures are indicated on the x-axis: The masked FC is the subset of FC features corresponding to the SC mask used for the MOU-EC model; “heur” corresponds to the symmetric connectivity matrix obtained using the heuristic optimization in Appendix D in the Supporting Information. The classifiers are trained with 80% of the subjects and tested on the remaining subjects, over 40 repetitions. The dashed line indicates the chance level, which corresponds to predicting always movie (three sessions out of five). The MLR gives perfect classification for all connectivity measures except for MAR. (C) Same as panel B for the classification of session to identify the four tasks (three movie sessions and rest). The chance level corresponds to predicting always rest (two sessions out of five). (D) The left confusion matrix indicates the errors of the MLR classifier trained with five labels (two labels for rest here), as indicated by the dark green off-diagonal pixels. As expected from panel B, rest and movie are well separated. The movie sessions (M1 to M3) are distinguishable to some extent, but not the rest sessions (R1 and R2). The right confusion matrix is the equivalent for the 1NN classifier. (E–F) Same as panels C–D for the identification of the 22 subjects. Here 40 splits of the data are considered for two sessions in the train set and three sessions in the test set, irrespective of rest/movie and individually for each subject. (G) Model error for the MOU-EC and heuristic optimizations in the two conditions.

Although machine-learning techniques are now increasingly used to analyze neuroimaging data, the generalization capabilities are not always properly verified (Varoquaux et al., 2017). For example, clustering algorithms applied on reduced-dimension components (see Figure 3B–C) give a measure for the separation of the tasks viewed using the connectivity measures (Figure 3D). Notice that all sessions are used for the dimensionality reduction in Figure 3. This is a problem since the parameters tuning of the model can be influenced by the specific noise of the data samples and, in turn, the results will be not generalizable to new samples—a phenomenon called overfitting. To evaluate the robustness of a classifier, a cross-validation procedure is the standard for voxel-wise studies for activation maps (Naselaris et al., 2011; Walther et al., 2016) and for clinical applications (Hohenfeld, Werner, & Reetz, 2018; Rahim et al., 2017). This procedure consists in splitting the data samples in train and test sets that are respectively used to fit the classifier and to assess its performance, as described in Figure 4A. Thus, one may have to include preprocessing steps in the cross-validation scheme to properly evaluate the generalization capability of the classification, for example with functional parcellations derived from the data that should be calculated on the train set only (Brodersen et al., 2011). For LDA in Figure 3C, this corresponds to setting a linear boundary using the dimensionality reduction on the train set and evaluating the performance on the test set. The data splitting can be repeated several times in order to assess the impact of different samples in the training and test set on the classification performance. Thus, the accuracy distribution provides an estimate of the generalization capability of the classification scheme to new samples. Different splitting strategies have been proposed, and here we use the recommended one for neuroimaging applications based on a ratio of 80%:20% for train and test sets, respectively (Varoquaux et al., 2017). We repeat the classification for 40 random splitting of the data. This is lower than the recommended 100 times and is justified by the rather low number of 22 subjects in our dataset, as explained in the box below together with further technical considerations about choosing a cross-validation scheme.

#### Remark on cross-validation schemes

The recommended ratio for neuroimaging applications is 80%:20% for train and test sets, respectively, chosen by random draws and repeated about 100 times (Varoquaux et al., 2017). While we agree on this as a good general practice, we highlight that a slightly different scheme might be better suited when the number of available samples is rather low, as is often the case in cognitive studies like here with 22 subjects. Another issue concerns the independence or absence thereof of the measurements used as inputs for classification. For the example of Figure 3, each subject is scanned in both resting-state and movie conditions, meaning that fMRI measurements are paired together. There, random resampling based on fMRI sessions without separating subjects in train and test sets would lead to more similar samples in the train and test sets than expected by chance. This would likely inflate the evaluated performance for generalization. A better solution is to apply the cross-validation to “groups” (subjects in this case) instead of single samples (fMRI sessions). While it is possible to repeat random resampling of the groups several times, the chance of repeating the cross-validation for the exact same test set becomes nonnegligible when the number of subjects is not sufficiently large. For our dataset, using four subjects for testing and the remaining 18 for training, there is almost 50% chance of getting the same split twice over 100 repetitions. The consequence would be an underestimation of performance variability. In such cases, an alternative valid option is the leave-one-group-out strategy, using a subject for testing and the remaining subjects for training, yielding a distribution of 22 accuracies. This procedure also has the advantage of highlighting individual differences in the dataset, showing for example whether some subjects are easier or more difficult to predict. With our data, we find that repeating 80%:20% shuffle splits 40 times and performing leave-one-out for each of the 22 subjects leads to very similar accuracy distributions.

We consider task discrimination in two flavors: movie versus rest (two tasks) in Figure 4B and the movie sessions individually plus rest (4 tasks) in Figure 4C. For each case, the performance evaluated using the test set should be considered relative to perfect accuracy and chance level (dashed line). As expected from Figure 3, the four-task discrimination is more difficult that the two-task one. The relative performance decreases by about a half of perfect accuracy for the four tasks compared with the two tasks. There are three interesting points coming from the comparison of the performances between the connectivity measures.

First, the MLR and LDA perform much better than the 1NN for the four-task discrimination, with both MOU-EC and FC; this does not change when using kNN instead of 1NN. This agrees with previous results on subject identification (Pallarés et al., 2018) that are transposed to the present dataset in Figure 4E. For task identification, the Pearson-correlation similarity for FC or MOU-EC does not seem the best choice, especially when the environment is complex. A technical point here is that LDA takes a long time to be trained for a large number of features or for many labels (subjects in Figure 4E). Over all these results, the MLR seems the best practical choice for a classifier, as was found for clinical data (Dadi et al., 2019).

Second, and more importantly, the masked FC performs worse than FC for the four tasks in Figure 4C, demonstrating that the SC mask (with 25% density here) cannot be directly used to preselect the most informative FC links that contribute to the good performance. In contrast, MOU-EC has the same number of features as the masked FC and performs as well as FC, even though the silhouette coefficient in Figure 3D is lower for MOU-EC than FC. Moreover, the MAR connectivity estimate, which extracts temporal information from BOLD signals as MOU-EC does, gives very poor accuracy, even for the two-task classification. Partial correlations are somewhat in between. For subject identification, MOU-EC performs better than FC in Figure 4E, in line with previous results (Pallarés et al., 2018). Taken together, these results show that the dynamic model used for MOU-EC is a representation of the BOLD signals that extracts relevant information for both cognitive conditions and individual traits. In terms of dimensionality, MOU-EC can be seen as a more compressed version of the BOLD information than FC without loss of information.

Last, we consider the symmetric connectivity obtained by the heuristic optimization in Appendix D (see the Supporting Information) that tunes the MOU model to reproduce the zero-lag correlation FC. In Figure 4G the goodness of fit is slightly better for the heuristic method (smaller model error measure by the matrix distance) compared with EC. It is worth noting that the heuristic estimate leads to perfect accuracy for the “simple” classification rest versus movie (Figure 4B). However, the accuracy decreases compared with MOU-EC for the four-task and subject classifications by 5% and 12%, respectively (Figure 4C, E). This shows that the choice for the connectivity estimation method (with the corresponding measure on the BOLD activity) is crucial to efficiently extract information from the BOLD signals.

### Capturing the Hierarchy of Cognitive States

The high dimensionality of connectivity measures allows for representing complex environments, such as the four tasks considered here. Beyond their ability to classify, the important question is whether MOU-EC or FC can capture the structure of the task categories—rest on one hand and the group of three movie sessions on the other. A similar goal was sought using clustering on FC to identify subtypes of depression (Drysdale et al., 2017), although concerns have been raised about the reproducibility of the results (Dinga et al., 2018). Clustering corresponds to unsupervised learning (without cognitive labels) and seeks a structure in the data for a given metric as with the first two components of PCA in Figure 3B.

Instead, we rely on supervised learning and propose labels as an informed guess to a classifier, then assess whether they are meaningful using cross-validation. Beyond the performances of the two-task and four-task classifications in Figure 4B–C, each classifier thus defines a similarity metric for the connectivity measures. This is reflected in the confusion matrices in Figure 4D, where the classification errors (dark off-diagonal green pixels) indicate which cognitive states are difficult to separate. By the naked eye, the structure of the left confusion matrix for the MLR determines a hierarchy that corresponds to the desired one, similar to the clustering in Figure 3B and C. When asked to discriminate between the two rest sessions, the classifier gives a negative answer. The three movie sessions are identifiable even though the MLR classifier makes errors between them. In contrast, the right confusion matrix for the 1NN does not provide useful information. Similar matrices without a clear hierarchy are obtained for FC (not shown). This suggests that the Pearson correlation used as a similarity measure in the 1NN is not well suited to describe the cognitive states with sufficient flexibility. In other words, it is less the global EC/FC profile than specific links that significantly vary across tasks and should be the basis for building the task hierarchy. We do not enter into further details here, leaving the comparison between unsupervised and supervised learning in estimating label hierarchies in more depth for future work. As a last comparison, the confusion matrix for subjects in Figure 4F shows that subjects are well identified and no particular structure in the errors is observed, indicating an absence of subject groups.

### Biomarker Formed of Informative MOU-EC Links: Machine Learning Versus Statistical Testing

**Figure 5.**
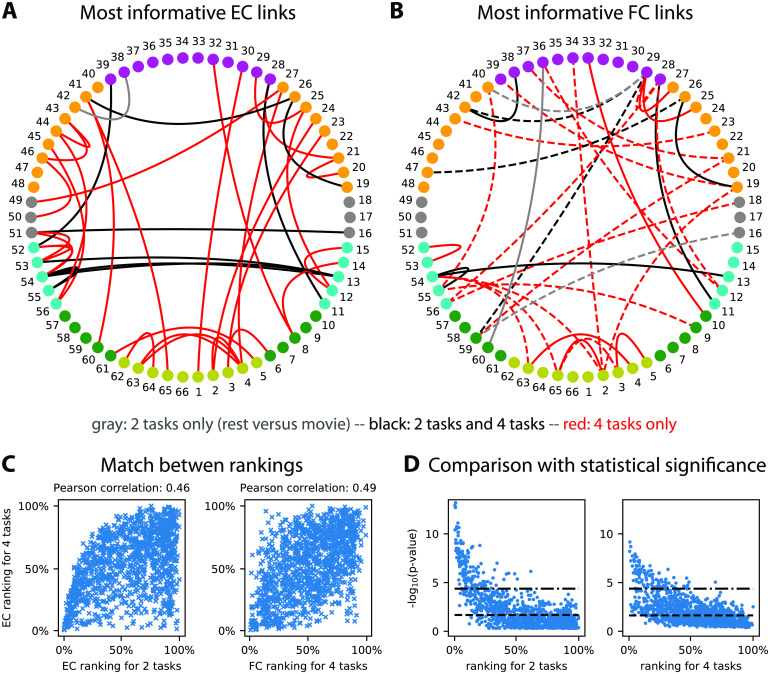
Biomarker for tasks and subjects. (A) Support network of the 35 most informative MOU-EC links for the task identification, obtained using recursive feature elimination (RFE). The 10 black links are common to the easier discrimination between rest and movie, whereas the 29 red links are specific to the four tasks, and the gray link is specific to the two tasks. The ROI colors indicate anatomical grouping: yellow = occipital, green = parietal, cyan = temporal, gray = central, orange = frontal, and purple = cingulate. See also Table A.1 for the ROI labels. (B) Same as panel A for FC links. We obtain eight common black links, three gray links for the two tasks only, and 21 red links for the four tasks only. The links in dashed lines correspond to ROI that are not connected in SC. (C) Match between EC rankings for the two and four tasks (left plot), as well as between EC and FC rankings for existing EC links. The Pearson correlation between the matched rankings is indicated above. (D) Correspondence between *p* values for Mann-Whitney tests and RFE rankings for the 1,180 MOU-EC links. For the two tasks, we show two significance thresholds for multiple comparisons: the Benjamini-Hochberg correction (dashed line) and Bonferroni correction equal to −log_10_(0.05/1180) (dashed-dotted line). For the four tasks, the maximum of the *p* values for the six pairwise comparisons is plotted, with the same Bonferroni threshold.

The general idea behind a biomarker is the indication of how and where to measure relevant information in the brain activity for the discrimination of several conditions, here related to cognition. The biomarkers for the two-task and four-task classifications in Figure 4B–C are built by identifying the most informative links that contribute to correct classification, also referred to as support network. Practically, we employ recursive feature elimination (RFE) that efficiently works with the MLR classifier (Guyon, Weston, Barnhill, & Vapnik, 2002). It provides a ranking of the features in order of importance, and the support network consists of the first ranked links (with a cutoff when the classification performance based on those selected links reaches a maximum). A previous study compared these support networks of informative links for subjects and tasks to see whether the two classifications were biased one by the other (Pallarés et al., 2018). Here we make another comparison with the two support networks in Figure 5A, movie versus rest (nine black and two gray links) and for the four tasks (nine black and 35 red links). The nine common links are also a hallmark of the hierarchy mentioned in the previous section, in the sense that the more four-task biomarker extends the two-task biomarker. Furthermore, the two RFE rankings are robustly aligned (Pearson correlation equal to 0.46 with *p* value ≪ 10^−10^), as illustrated in Figure 5C (left plot). If we compare with the support network for the FC links in Figure 5B, we observe a similar overlap between the two support networks. Importantly, five FC links out of the 12 for the two-task classification do not correspond to anatomical connections in SC, and 20 out of 30 for the four-task classification. This suggests that FC changes reflect network-wise consequences rather than modulations of anatomical connections between ROIs (in dashed line). Nonetheless, we find that the RFE rankings for EC and FC are correlated, with a Pearson coefficient of 0.49 (and *p* value ≪ 10^−10^); see the right plot in Figure 5C.

Now we discuss the pros and cons of machine learning compared with statistical testing in finding correlates (i.e., link changes) of cognitive tasks. Formally, these two methods correspond to distinct questions that relate to opposite mappings from measured variables to conditions: changes in the variables across conditions for statistical testing and the prediction of conditions based on the variables for machine learning. However, they can be used to address the same practical question, especially when interpreting data in two conditions (as here with rest versus movie). In neuroimaging data a crucial point relates to the high dimensionality of connectivity measures, which leads to repeated hypothesis testing—are MOU-EC values different across two conditions?—over the 1,180 connections. This calls for correction for multiple comparisons, which is problematic as the number of connections scales with the square of the number of ROIs in general. We illustrate this point by calculating the *p* values for the nonparametric Mann-Whitney test between rest and movie MOU-EC weights for each connection (left plot in Figure 5D). To identify links that strongly differ across conditions, we use the significance threshold with Bonferroni correction (dashed-dotted line), here −log_10_(0.05/1180) for 1,180 MOU-EC links, or better with Benjamini-Hochberg correction (Benjamini & Hochberg, 1995; dashed line). The same can be done for the four tasks (right plot), which involves six pairwise tests between the four cognitive states. For each connection we retain the smallest *p* value among the six (as it is informative for at least a pairwise discrimination). For the Bonferroni correction, this gives 98 connections passing the significance threshold for the four-task, to be compared with 153 for two tasks. Using Benjamini-Hochberg correction, we find for the two classifications 554 and 498 links, respectively. This does not satisfactorily reflect the increase of complexity of the cognitive labels—more labels require more connections in the biomarker—while the support network obtained using RFE in Figure 5A provides a satisfactory description. More importantly, the comparison of the mapping between *p* values and the RFE ranking in Figure 5D also illustrates that efficient classification may rely on links with poor *p* values, and that this phenomenon may be stronger when the number of categories increases (like labels here). This conclusion also holds when using the parametric Welch *t* test instead of the nonparametric Mann-Whitney test. Beyond the details of the present example, machine learning seems better suited than statistical testing to deal with connectivity measures in a context of multiple labels.

Another conceptual difference between the two approaches is the Gaussian assumption for the parameter distribution. For example, DCM relies in many cases on a single generative model with a distribution for each parameters—typically determined by a mean and a variance for each connection weight—and selects the model that provides the best evidence for all observed FC matrices. Instead, we estimate a single MOU-EC matrix per session, which builds a distribution of point samples for each connection. It remains to explore the implications of the difference in the natures of the estimate distributions and the detection of significant changes in MOU-EC—using the Bayesian machinery for DCM and standard machine learning in our case.

## NETWORK-ORIENTED ANALYSIS FOR INTERPRETING COLLECTIVE BOLD DYNAMICS ACROSS CONDITIONS

So far, we have shown how MOU-EC estimates can be used for the classification of cognitive conditions using machine learning. Now we turn to the collective interpretation of MOU-EC links as a network. Tools from graph theory have been extensively applied to explore the brain network and understand how cognitive functions were distributed over subnetworks of ROIs. The study of both FC and SC has revealed a hierarchical organization of modular networks that are interconnected by hubs (Hilgetag & Grant, 2010; van den Heuvel & Hulshoff Pol, 2010; Zamora-López, Zhou, & Kurths, 2011), which can be modulated depending on the subject’s condition (Bertolero, Yeo, & D’Esposito, 2015; Meunier et al., 2012; Senden, Goebel, & Deco, 2012).

A limitation in many SC and FC studies comes from the direct application of graph measures defined for binary data. In such cases SC or FC matrices are often binarized using an arbitrary threshold to obtain a graph, discarding the information conveyed by the graded values. For SC, this gives the skeleton of strong connections. Similar methods have also been used on FC (de Pasquale et al., 2012; Fair et al., 2007), but the use of a threshold to binarize FC seems more arbitrary, especially as many pairs of brain regions exhibit strong correlation. Another important aspect is often overlooked: Brain connectivity measures such as FC are inferred from signals that have a temporal structure. This means that network analysis should take time into account. A possibility is to analyze the evolution of snapshot networks over time using dynamic FC based on sliding windows applied to the observed BOLD signals (Avena-Koenigsberger, Misic, & Sporns, 2017; Khambhati, Sizemore, Betzel, & Bassett, 2017; Thompson, Brantefors, & Fransson, 2017).

Going a step further, a particular focus has been on studying the relationship between the SC and FC (Bettinardi et al., 2017; Sporns, Tononi, & Edelman, 2000), in particular using dynamic models at the mesoscopic or macroscopic level (Breakspear, 2017; Deco et al., 2011; Frässle et al., 2018; Galán, 2008; C. J. Honey, Kötter, Breakspear, & Sporns, 2007; Messé et al., 2014; Park & Friston, 2013; Schmidt, Bakker, Hilgetag, Diesmann, & van Albada, 2018). Following, graph analysis can be performed on the modeled BOLD activity (Deco & Kringelbach, 2017; Fransson, Schiffler, & Thompson, 2018). Nonetheless, it can be argued that such “integration” measures somehow rely on the observed phenomena (in empirical or model activity) rather than their causes. Rather, dynamic models enable the application of graph measures to the underlying connectivity that generates the dynamics, as was recently proposed (Gilson et al., 2019; Razi et al., 2017).

Here we follow this novel direction with our adaptation of graph-like measures for the network dynamics themselves (Gilson et al., 2019; Gilson, Kouvaris, et al., 2018), whose main details are presented in the following; see also Appendix E in the Supporting Information for mathematical details. This section illustrates the following aspects of network-oriented analysis for MOU-EC:

•  How can we interpret collective changes in MOU-EC in their contribution to shape the network dynamics? We introduce dynamic communicability with the notion of “integration time” that discriminates early and late interactions between ROIs in response to a (local) perturbation in the network via MOU-EC.
•  Are network measures relevant for discriminating cognitive conditions? By doing so we want to obtain dynamics-specific biomarkers.
•  Between the level of individual connections and the global network, are intermediate scales relevant to interpret the MOU-EC estimates? Can we detect functional communities of ROIs, and are they task-specific?

### Dynamic Flow as Graph-Like Measure for Interactions Between ROIs Across Integration Time

**Figure 6.**
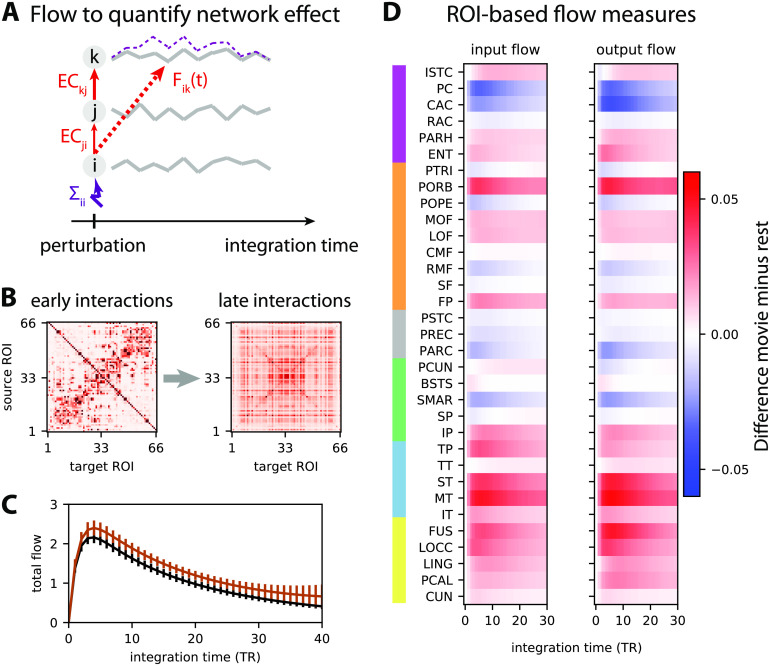
Network-oriented analysis of MOU-EC using dynamic flow. (A) Conceptual illustration of dynamic communicability and flow. The propagation of extra activity resulting from a perturbation (here applied to ROI *i* in purple) develops over the integration time and then fades away. The dashed purple curve represents the corresponding activity increase for ROI *k* (for legibility purpose, the equivalent for ROI *j* is not displayed). This defines interactions between ROIs that are not directly connected, like here from ROI *i* to ROI *k*. The dynamic communicability corresponds to interactions determined by the connectivity MOU-EC only, while the dynamic flow incorporates the amplitudes of ongoing perturbations that is quantified by Σ in the model. (B) Two matrices representing the pairwise interactions as measured by dynamic communicability for all 66 ROIs at two integration times, in a single subject. Initially similar to MOU-EC values, the communicability becomes homogeneous. (C) Total flow for rest and movie, which is the sum of the interactions between all pairs of ROIs (i.e., sum of all matrix elements in panel B) at each integration time. The error bars indicate the standard error of the mean over the subjects. The bottom brown horizontal line indicates where the total flow is larger for movie than for rest with *p* < 0.01 in a Mann-Whitney test for each integration time step (without correction). (D) Change in input and output flow as a function of integration time, which sum all incoming and outgoing flow interactions for each ROI, respectively. The ROIs are ordered by anatomical areas and grouped in pairs of left- and right-hemisphere ROIs. The ROIs are anatomically grouped in the same manner as in Figure 5A–B: yellow = occipital, cyan = temporal, green = parietal, gray = central, orange = frontal, and purple = cingulate from bottom to top.

Our definition of *dynamic flow* takes advantage of the information provided by the dynamic model, here applied to the MOU process (Gilson, Kouvaris, et al., 2018). As illustrated in Figure 6A, MOU-EC quantifies the instantaneous causal interactions between pairs of ROIs, and the Σ matrix measures the spontaneous or input activity level that each ROI receives (here represented only for ROI *i*). Built on those estimates, the dynamic flow measures the influence of a “perturbation” at a source ROI *i* onto a target ROI *k* after a given delay, which we call integration time. Importantly, it takes into account the network effect (or network response), meaning the contribution of indirect paths in the network (from ROI *i* to ROI *k* via ROI *j* in the figure). See Appendix E in the Supporting Information for the mathematical formulation based on the network impulse response (or Green function), which is analytically tractable for the MOU process.

The dynamic flow thus aims to capture the dynamic response of the brain to local spontaneous activity (or external inputs) as it propagates from region to region in the network. A special case of the dynamic flow—termed *dynamic communicability*—that only depends on the MOU-EC (and not Σ) can also be defined assuming that all ROIs have independent spontaneous activity with the same intensity; it corresponds to a diagonal and homogeneous matrix Σ. Like the flow, dynamic communicability describes directional interactions, which differs from the symmetric interactions obtained from the direct application of graph theory to FC or SC (Gilson et al., 2019). It is worth stressing that the dynamic flow and communicability are graph-like measures that genuinely capture the properties of weighted networks, similar to the previously proposed graph communicability (Estrada, 2013; Estrada & Hatano, 2008).

Considering again the fMRI dataset in Appendix A in the Supporting Information, we examine the dynamic flow at various scales in the brain network for the two conditions, movie and rest. This complements our previous analysis of the same dataset that showed that the modulations of MOU-EC between rest and movie implemented a selection of pathways in the cortex (Gilson, Deco, et al., 2018). In addition, the stimulus load related to sensory inputs in the movie condition corresponded to an increase in Σ in the model for occipital and temporal ROIs. Recall that Σ quantifies the fluctuating BOLD activity intrinsic to each ROI that subsequently propagates via MOU-EC in the model. This motivates the use of the dynamic flow that incorporates Σ in order to characterize the whole-brain BOLD propagation, as compared with dynamic communicability. Note that the “effective drive” defined in our previous study (Gilson, Deco, et al., 2018) corresponds to the flow at early integration time.

The sum of flow interactions provides insight about how much activity propagates throughout the whole cortical network, which is higher for movie compared with rest in Figure 6C. Analyzing the interactions at the ROI level, the flow changes in Figure 6D indicate differentiated effects among the ROIs, with increased/decreased listening for input flow and broadcasting for output flow. Here we observe most of the strongest increases in the output flow for occipital and temporal ROIs, which makes sense since the subjects both watch and listen to the movie. A previous analysis based on MOU-EC (Senden et al., 2017) showed task-dependent changes for the outgoing connections from the rich club of ROIs that have dense anatomical connectivity, namely the precuneus (PCUN), superior parietal cortex (SP), and superior frontal cortex (SF), in agreement with previous results about the default mode network also involving the post-cingulate gyrus (PC Vatansever, Menon, Manktelow, Sahakian, & Stamatakis, 2015; Vatansever, Menon, & Stamatakis, 2017). Here we only find a slight increase of input flow for PCUN and SP, which may be explained by the fact that the task is passive viewing and listening.

### Integration in the Brain network

**Figure 7.**
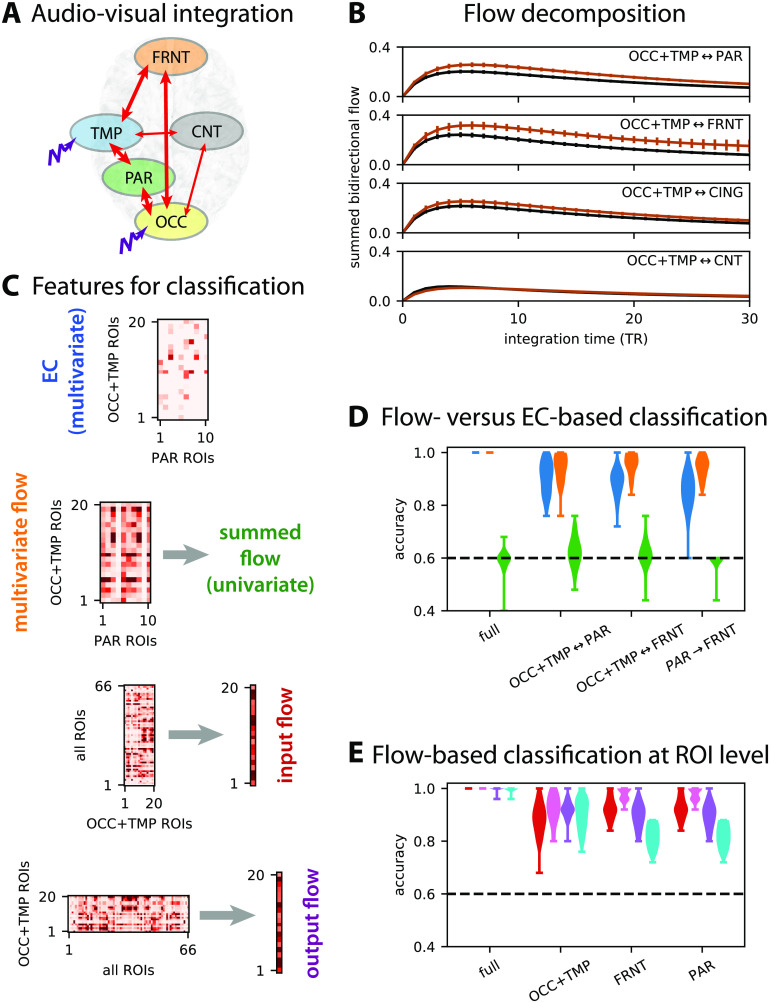
Integration measured by dynamic flow. (A) Example subset of anatomical ROI groups: occipital (OCC), temporal (TMP), parietal (PAR), central (CNT), and frontal (FRNT). (B) Comparison between the flow between the rest (in black) and movie (in brown) conditions. Similar to Figure 6C, the curves are averages over subjects and corresponding sessions with error bars indicating the standard error of the mean. Each row corresponds to the flow summed over all ROIs of the indicated groups (bidirectionally); see also panel A where thick arrows correspond to strong increase in flow for the movie condition. (C) Example of construction of features for the following classification. At the top, the EC matrix corresponds to connections for ROIs in groups and gives multivariate features when vectorized. Below, the flow matrix for the same ROIs equivalently gives multivariate features or can also be summed to obtain a univariate feature. Moreover, the flow matrix can be summed over columns or rows to obtain the input and output flows, here taking all 66 ROIs as sources (y-axis of the matrix) or targets (x-axis). (D) Flow-based classification to discriminate rest versus movie in multivariate (orange violin plots) and univariate (green), compared with EC-based classification (blue). The x-axis indicates the ROI subgroups for which the flow interactions are selected. The dashed black lines indicate chance level, at 60% as in Figure 4B. (E) Same as panel D for the input flow (in red) and output flow (purple), which are compared with the input and output EC strengths (pink and cyan, respectively).

Following experiments on the propagation of stimulus information from sensory areas to “high-level” brain areas in relation to being conscious of the stimuli (Carhart-Harris et al., 2016; Dehaene & Naccache, 2001), the concept of integration was defined in models by quantifying the influence between regions while being in a broader network. Following Figure 6D, we use the dynamic flow to quantify how the BOLD activity propagates from the occipital and temporal ROIs, which exhibit an increased visual and auditory stimulus load in movie, to the rest of the brain. We thus consider the anatomical ROI groups in Figure 7A (same as Figure 5), where the stimulus load is represented by the purple perturbation applied to OCC and TMP (Gilson, Deco, et al., 2018). Figure 7B compares the summed flow between the ROI groups for rest and movie (in black and brown as in Figure 6C). This reveals preferred pathways to the parietal and frontal ROIs (PAR and FRNT) when sensory information is integrated, as represented by the thick arrows in Figure 7A.

The machine-learning equivalent of the statistical testing for the summed flow between subnetworks in Figure 7B gives the green violin plots in Figure 7D. Here a good accuracy means that the bidirectional flow summed between all considered ROIs is strongly modulated between rest and movie. The fact that the results are close to chance level indicates strong variability in the overall flow increase across subjects. A different question, more related to biomarkers, is to which extent the detailed pattern of flow (multivariate flow, in orange) vary between movie and rest. This yields robust classification, which strikingly differs from the univariate (summed) flow. This indicates that the modification of interactions between ROIs is diverse beyond a simple global increase, hinting at the selection of specific pathways. The flow-based classification even outperforms MOU-EC (in blue) for the same matrix elements when focusing on particular ROI groups; see Figure 7C for details. The discrepancy between the flow and MOU-EC shows the importance of network effects, for all three bidirectional pathways in Figure 7D. The same classification can be performed with the input and output flows; see Figure 7E. For interpretation purpose, the better performance for incoming than outgoing MOU-EC (pink versus cyan) suggests that ROIs change their pattern of listening/broadcasting communication, especially for PAR and FRNT. However, the overall effect is less pronounced when considering the dynamic flow that incorporates network effects (red versus purple). These results show that dynamics-based biomarkers that describe the propagation of BOLD activity can be built in the same way as connectivity-based biomarkers for the discrimination of cognitive tasks. Together with the distributed signature found in Figure 5A, they also highlight the need for a whole-brain approach to understand the cortical reconfiguration (Betti et al., 2013), contrasting with previous studies relying on hypothesis testing for a priori selected ROIs (Ciuciu et al., 2014; Daunizeau, Stephan, & Friston, 2012; Emerson, Short, Lin, Gilmore, & Gao, 2015; Goebel et al., 2003).

### Detection of Functional Communities Determined by Dynamic Flow

In parallel to integration, the concept of complexity has been often used to differentiate brain states (Barnett et al., 2009; Tononi et al., 1994; Tononi, Edelman, & Sporns, 1998; Zamora-López, Chen, Deco, Kringelbach, & Zhou, 2016). Intuitively, complexity measures quantify the diversity of organizational motifs in a network, in the range of interest between the trivial extremes of full disconnectivity and full connectivity. A proxy for complexity is the detection of ROI communities because modular networks are a stereotypical model for complex networks, as was observed with SC (Zamora-López et al., 2011). Here we use the dynamic flow as a basis for the functional ROI communities in a data-driven manner, which contrasts with the anatomical grouping in Figure 7A. Such methods developed initially for graphs with a metric called modularity based on weights (Newman, 2006). Here we consider communities determined by the flow, namely ROIs with strong exchange of activity in the estimated dynamics (Gilson, Kouvaris, et al., 2018).

**Figure 8.**
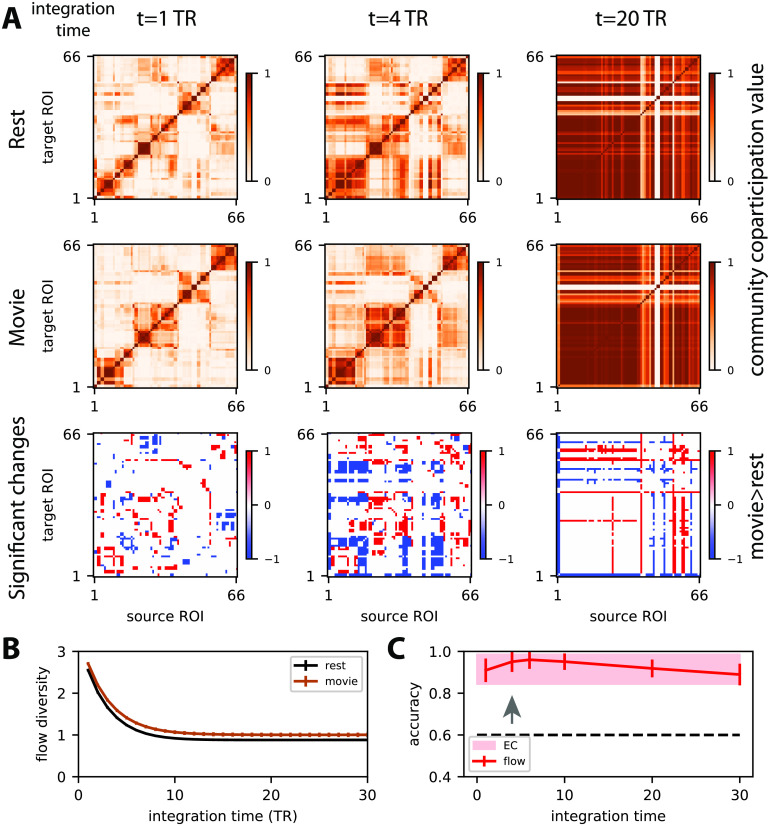
Community detection. (A) Communities correspond to ROIs that bidirectionally exchange strong flow. Darker orange pixels indicate stable communities across the subjects for each condition. The top row corresponds to rest and the middle row to movie for three integration times. The bottom row displays the significant increases (in red) and decreases (blue) of the community coparticipation values for movie with respect to rest. Here statistical testing is performed on each coparticipation value by using the Mann-Whitney test with *p* < 0.01 (without correction). The ROI ordering corresponds to the early community structure for rest. (B) Flow diversity for rest (black curve) and movie (in brown) measuring the heterogeneity within each flow matrix when integration time increases; see Equation E.4. It is a proxy for the “complexity” of interactions between ROIs; for details see Gilson et al. (2019) and Gilson, Kouvaris, et al. (2018). (C) Rest-versus-movie classification based on flow (whole-brain version of multivariate flow in Figure 7C) as a function of integration time and comparison with EC-based classification. Starting *t* = 4 TRs indicated by the arrow, the performance reaches a maximum plateau. Here the train test consists of 20% of the samples and the test set 80% (the converse compared with before) in order to capture the strongest changes between rest and movie with classification. This exaggerates the evolution over integration time of the performance, which is lower compared with the previous plots.

Figure 8A displays the evolution of communities over integration time for rest and movie. Strong coparticipation values indicate the robustness of the community structure across subjects. It shows a merging of almost all ROIs that eventually exchange flow in an even manner. This agrees with our previous results for resting state only and a different parcellation (Gilson et al., 2019). This speaks to a transition from segregated processing (within each diagonal block of left matrices) to a global integration. The number of communities can be taken as another proxy for the complexity in the brain network, and our approach provides a dynamical perspective about it. The community merging goes hand in hand with the homogenization of the flow matrix in Figure 8B, as quantified by the diversity of the matrix elements in the flow matrix that decreases and eventually stabilizes (Gilson, Kouvaris, et al., 2018). In our data the diversity is slightly higher for movie compared with rest.

Going a step further, we can examine the difference between the community structures of the two conditions; see the bottom row in Figure 8A. This shows that the changes in dynamic flow for specific pairs of ROIs between rest and movie (Figure 7B, D, and E) have an important collective impact. The strongest differences appear when the network effects are very strong at *t* = 4 TRs. This is confirmed by Figure 8C, where classification shows that the dynamic flow is most different between rest and movie at integration times corresponding to strong network effects (the arrow indicates the start of the plateau for maximum performance). These results speak to an intermediate organization of groups of ROIs between the local and global scales, which can be captured using our network-oriented analysis of MOU-EC.

## CONCLUSIONS

This article illustrates how a model-based approach to whole-brain fMRI analysis combines the desired properties of predictability and interpretability. This goes beyond the goodness of fit for fMRI signals that is commonly used to evaluate and compare generative models. To do so, our framework links tools and concepts from dynamic systems, machine learning, and network theory. This results in a consistent pipeline with controlled hypotheses because we use the same whole-brain dynamic model from the estimation to the analysis.

**Table:** General key points for analysis of multivariate neuroimaging data and corresponding results using MOU-EC

General key points	Presented results using MOU-EC
A dynamic model is a hypothesis on the structure of the data and is useful to combine several types of data.	The MOU process provides a practically useful balance between the richness of the generated BOLD dynamics, the robustness of the model optimization, and intuition for interpretability (roles of parameters in the model). The constraint of MOU-EC topology enforces the “explanation” of changes in BOLD signals by modulations of anatomical connections (Figure 5A compared with Figure 5B).
Fitting a dynamic network model to time series captures their dynamics and enables the characterization of network properties with time involved in the analysis.	MOU-EC describes causal relationships in the network model that collectively explain the observed BOLD covariance structure, involving time lags. The MOU-EC estimates can further be interpreted in terms of integration in the network using dynamic communicability and flow (Figure 6).
Adequate machine-learning tools (with cross-validation and feature selection to build biomarkers) are well adapted to relate multivariate neuroimaging data like fMRI to cognitive states or neuropathologies.	Both MOU-EC and dynamic flow can be used for prediction (Figures 4B–C, 5A, and 7D–E), as well as exploring the hierarchy of cognitive states (Figure 4D).
Graph-like measures can be applied at multiple scales from the connection/node level to communities and global level in the network.	The comparison of classification performance for tasks using dynamic flow provides insight about the adequate description level of changes in the network; for example, ROI-based measures are sufficient to discriminate the rest and movie conditions (Figure 7D–E).

### Spatiotemporal BOLD Structure

By fitting the dynamic model to the spatiotemporal covariance structure of the BOLD signals, our approach captures the BOLD dynamics averaged over each fMRI session. The estimated MOU-EC can be seen as a “projection” of the BOLD signals on anatomically constrained connectivity, thereby characterizing the dynamical state of the brain. In contrast, analyses based on the static FC can be related to a linear Gaussian model, and thus do not take the temporal dimension into account (i.e., BOLD values are considered as i.i.d. variables). Dynamic FC methods have been developed to incorporate time in the analysis (Cabral et al., 2017; Gonzalez-Castillo & Bandettini, 2017; Park & Friston, 2013; Preti et al., 2017), but they do not genuinely capture the propagating nature of BOLD signals. Dynamic causal models have just been adapted to deal with whole-brain fMRI data (Frässle et al., 2018; Park, Friston, Pae, Park, & Razi, 2018; Razi et al., 2017), and it will be interesting to compare the estimated connectivities. It is also still unclear how short sessions or observation windows can be to robustly characterize dynamical states when calculating EC estimates or MAR-like coupling matrices in HMMs (Bolton et al., 2018; Vidaurre et al., 2018), in comparison to dynamic FC methods that are typically used with windows of 30 to 60 TRs.

### Biomarkers for Cognition and Neuropathologies

The robust estimation of the (multivariate) parameters allows for a powerful representation of cognitive states by the MOU-EC estimates (Figure 4), as well as the extraction of task-specific biomarkers (Figure 5). An important point considering connectivity measures is that machine learning solves the problem of multiple comparisons: Instead of determining *p* values and then a threshold for connections with significant changes, we can characterize which MOU-EC links—as features—contribute to the correct classification. It has been recently advocated that machine learning can be used to avoid “excessive reductionism,” namely linking brain activity in localized regions to narrow experimental protocols in a way that cannot generalize to more complex protocols (Varoquaux & Poldrack, 2019). Although the focus has been on machine-learning techniques, we remind readers that hypothesis testing of significant changes for preselected ROIs or links can be performed in our framework (see Figure 7B with the dynamic flow). In that case, whole-brain changes can also provide a baseline reference for the considered links or ROIs.

On the technical side, it remains to be explored how the classification performance is affected by the resolution of various parcellations (Craddock, James, Holtzheimer, Hu, & Mayberg, 2012; Eickhoff, Thirion, Varoquaux, & Bzdok, 2015; Glasser et al., 2016), as was recently done with FC-like connectivity measures (Dadi et al., 2019). The trade-off to solve is between the richness of the connectivity measure (more links can reproduce richer environments) and their robustness (more parameters may result in noisier estimation). The alignment of distinct datasets with various MRI protocols also remains a difficult question for practical application to large datasets (Varoquaux et al., 2018).

In parallel to the study of cognition, the presented framework can be applied to neuropathologies with the aim to inform clinical diagnosis (Matthews & Hampshire, 2016). The rationale is that BOLD activity specifically reflects neuropathologies, even in the resting state (Greicius, 2008; Hohenfeld et al., 2018). If SC is increasingly used for strokes or Alzheimer’s disease that strongly impact the brain anatomy, fMRI may provide additional information (Habib et al., 2017; Siegel et al., 2016). Other diseases like depression (Drysdale et al., 2017) or some motor disorders (Rowe & Siebner, 2012) may be better investigated using functional imaging. Importantly, many neural diseases are likely to affect multiple brain regions and thus require adequate tools to capture their distributed nature. Another direction where machine learning is a key tool is the development of personalized medicine, adapting the models to individual patients beyond group analyses (Yahata, Kasai, & Kawato, 2017).

### Network-Oriented Analysis of Dynamics

**Figure 9.**
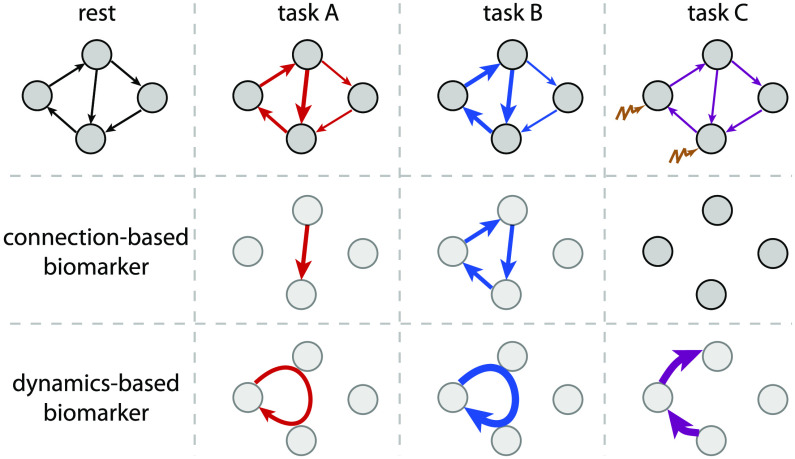
Schematic representation of the extraction of biomarkers for three tasks compared with rest. Connection-wise biomarkers correspond to changes in EC, while the bottom row refers to biomarkers for changes in dynamic flow. Tasks A and B correspond to two modulations of the connectivity, which lead to similar changes in the dynamic flow (stronger for B). For task C, extra inputs are received by two nodes, which increase the dynamic flow towards the corresponding two target nodes.

The network analysis for our dynamic model relies on the dynamic flow, a graph-like connectivity measure that describes the integration of propagating activity at multiple scales in the network (Figures 7 and 8). In particular, small changes in EC that are not individually significant can collectively induce large changes in the dynamic flow, especially when feedback loops are involved. In this sense, the dynamic flow quantitatively captures the interplay between parameters, so the resulting communication cannot always be simply understood at the level of single estimated parameters. As an example, many small changes in EC (below significance level) may collectively result in a strong dynamical effect, as seen for Task A in Figure 9. Strong coordinated changes in EC result in large changes in the dynamics (Task B). Moreover, changes in input properties may only be captured by the dynamics, in particular when the connectivity does not change (Task C). Building biomarkers that capture network effects is important to make use of the multivariate nature of the fMRI data. This is important when interpreting data in terms of concepts such as integration, segregation, and complexity (Deco, Tononi, Boly, & Kringelbach, 2015; Dehaene & Naccache, 2001; Tononi et al., 1998; Zamora-López et al., 2016). An interesting direction for future work is the study of directional properties of the flow, especially in the characterization of functional communities.

### Limitations and Future Work

As a first warning, we remind readers that any interpretation of BOLD in terms of brain communication relies on the assumption that changes in neuronal activity are reliably reflected in the BOLD signals, which is still under debate (Goense & Logothetis, 2008; Y. He et al., 2018; Matsui et al., 2018). In practice, the preprocessing of fMRI signals must strive to increase the signal-to-noise ratio (Parkes et al., 2018; Power et al., 2018).

Recall also that causal relationships between brain regions come from the directed connectivity in the model, whose “best” parameterization is estimated from the data. The estimation procedure is crucial, in particular for the dynamic flow here since the asymmetry of MOU-EC strongly affects the resulting activity propagation in the model. Moreover, without a proper experimental interaction with the brain activity like using stimulation, the notion of causality is subject to the initial model assumption (Hughes, 1997; Kaplan, 2011). This remark also applies to other methods for directed connectivity, such as Granger causality and DCM (Valdes-Sosa et al., 2011).

A key distinction of our approach compared with DCM is that our MOU-EC (or FC) estimates are calculated for each fMRI session, so we obtain a sample distribution for connection weight over all subjects for each condition (e.g., task). In contrast, DCM usually considers a single model per condition that aims to represent the variability over subjects with a parametric distribution (mean and variance) for each connection weight. The limitations and advantages of both approaches in terms of statistical power and generalizability remain to be practically compared, especially for large datasets.

Regarding the hemodynamics and explicit HRF modeling discussed at the end of the section presenting the MOU model, a quantitative comparison between MOU-EC, DCM, and Granger causality analysis to extract biomarkers or interpret fMRI data in terms of neuronal coupling is necessary in the future. A particular point concerns the observed heterogeneity of HRF across different brain regions and how it affects the estimated connectivity and its directionality (Handwerker, Ollinger, & D’Esposito, 2004). Previous works also suggest that the HRF is important for the reliability of connectivity analysis (Gitelman et al., 2003; Olszowy et al., 2019). This may increase the number of parameters in the model, so the robustness of the estimation should be verified using both forward modeling in synthetic whole-brain networks (Smith et al., 2011) and with classification from real data with known categories (e.g., subjects with several sessions each). A first step could be to extend the MOU-EC as a state-space model with a first-order filter for HRF (Sauvage et al., 2017).

## SUPPORTING INFORMATION

Supporting information for this article is available at https://doi.org/10.1162/netn_a_00117. The analysis tools have been organized in a package written in the open-source language Python: https://github.com/mb-BCA/pyMOU (Gilson, Zamora-López, & Insabato, 2019) for the MOU-EC estimation and https://github.com/mb-BCA/NetDynFlow (Zamora-López & Gilson, 2019) for dynamic communicability and flow. The code to reproduce some figures in this paper using data available on https://github.com/mb-BCA/notebooks_review2019. The classification uses the library scikit-learn (Abraham et al., 2014).

## AUTHOR CONTRIBUTIONS

Matthieu Gilson: Conceptualization; Methodology; Software; Writing - Original Draft. Gorka Zamora-López: Conceptualization; Methodology; Software; Writing - Original Draft. Vicente Pallars: Conceptualization; Methodology; Software; Writing - Original Draft. Mohit H. Adhikari: Conceptualization; Methodology; Software; Writing - Original Draft. Mario Senden: Conceptualization; Methodology; Software; Writing - Original Draft. Adrià Tauste Campo: Conceptualization; Methodology; Software; Writing - Original Draft. Dante Mantini: Resources; Writing - Review & Editing. Maurizio Corbetta: Resources; Writing - Review & Editing. Gustavo Deco: Funding acquisition; Writing - Review & Editing. Andrea Insabato: Conceptualization; Methodology; Software; Writing - Original Draft.

## FUNDING INFORMATION

Mario Senden, Horizon 2020 Framework Programme (http://dx.doi.org/10.13039/100010661), Award ID: Human Brain Project SGA2 No. 785907. Gorka Zamora-López, Horizon 2020 Framework Programme (http://dx.doi.org/10.13039/100010661), Award ID: Human Brain Project SGA2 No. 785907. Matthieu Gilson, Horizon 2020 Framework Programme, Award ID: Human Brain Project SGA2 No. 785907. Gustavo Deco, Horizon 2020 Framework Programme (http://dx.doi.org/10.13039/100010661), Award ID: Human Brain Project SGA2 No. 785907. Andrea Insabato, H2020 Marie Skłodowska-Curie Actions (http://dx.doi.org/10.13039/100010665), Award ID: MSCA grant agreement No. 841684. Gustavo Deco, Agencia Estatal de Investigación (http://dx.doi.org/10.13039/501100011033), Award ID: PSI2016-75688-P. Gustavo Deco, Consell Català de Recercai Innovació (http://dx.doi.org/10.13039/501100002810), Award ID: AGAUR Programme 2017 899 SGR 1545. Maurizio Corbetta, Italian Ministry of Research (MIUR), Award ID: Progetto Dipartimenti di Eccellenza Neuro-DiP. Maurizio Corbetta, Horizon 2020 Framework Programme (http://dx.doi.org/10.13039/100010661), Award ID: FLAG-ERA JTC.
