# Supplementary material for: Model-based whole-brain effective connectivity to study distributed cognition in health and disease
Source: Netw Neurosci. 2020 Apr 1;4(2):338–73. doi: 10.1162/netn_a_00117 (PMC7286310; doi:10.1162/netn_a_00117)
Supplement: Supplementary file 1 [file netn-04-338-s001.pdf]

Gilson, M., Zamora-López, G., Pallarés, V., Adhikari, M. H., Senden, M., Tauste, A…Insabato, A. (2020). Supporting information for "Model-based whole-brain effective connectivity to study distributed cognition in health and disease." *Network Neuroscience*, *4*(2), 338–373. https://doi.org/10.1162/netn_a_00117

Model-based whole-brain effective connectivity to study distributed cognition in health and disease

—

Supplementary Material

Matthieu Gilson*[1], Gorka Zamora-López[1], Vicente Pallarés[1], Mohit H Adhikari[1], Mario Senden[2], Adrià Tauste Campo[3], Dante Mantini[4,5], Maurizio Corbetta[6,7], Gustavo Deco[1,8], and Andrea Insabato[9]

[1]Center for Brain and Cognition and Department of Information and Communication Technologies, Universitat Pompeu Fabra, Barcelona, Spain
[2]Department of Cognitive Neuroscience, University of Maastricht, Maastricht, Netherlands
[3]BarcelonaBeta, Barcelona, Spain
[4]Neuroplasticity and Motor Control Research Group, KU Leuven, Leuven, Belgium
[5]Brain Imaging and Neural Dynamics Research Group, IRCCS San Camillo Hospital, Venice, Italy
[6]Department of Neuroscience, Venetian Institute of Molecular Medicine (VIMM) and Padova Neuroscience Center (PNC), University of Padua, Italy
[7]Department of Neurology, Radiology and Neuroscience Washington University School of Medicine, St. Louis, USA
[8]Institució Catalana de la Recerca i Estudis Avançats (ICREA), Barcelona, Spain
[9]Institut de Neurosciences de la Timone, CNRS, Marseille, France

The following sections summarize details about the data and mathematical framework used in the main text of the article. For machine learning, the readers are referred to the online documentation `http://scikit-learn.org/` of the scikit-learn library (Abraham et al., 2014).

*matthieu.gilson@upf.edu

A Movie dataset

The experimental procedure was approved by the Ethics Committee of the Chieti University and the participants signed a written informed consent. We use the data from 22 right-handed young and healthy volunteers (15 females, 20-31 years old) that had recordings for both a resting state with eyes opened and a natural viewing condition (30 minutes of the movie 'The Good, the Bad and the Ugly').

The fMRI time resolution (TR) is 2 seconds and each session of 10 minutes corresponds to 300 frames (or volumes). The preprocessing performed using SPM8 involves the coregistration of structural and functional MRI, spatial normalization in the MNI coordinates and motion correction. The data are parcellated in $N = 66$ ROIs (Hagmann et al., 2008). The ROIs are listed in Table 1, indexed in the order of Figs. 5 and 6 in the main text. The generic structural matrix is obtained from averaging individual matrices obtained using the diffusion spectrum imaging. A threshold was then applied to retain the 27% largest connections. Inter-hemispheric connections between homotopic ROIs were added, increasing the density to 28%. The same generic SC matrix is used to determine the MOU-EC topology for all subjects.

We refer the readers to previous publications (Hlinka et al., 2011; Mantini et al., 2012; Gilson et al., 2018) for further details.

B Functional connectivity measures

For each session of $T = 300$ time points (2 for rest and 3 for movie), we denote the BOLD time series by s_i^t for each region $1 \leq i \leq N$ with time indexed by $1 \leq t \leq T$. The mean signal is $\bar{s}_i = \frac{1}{T}\sum_t s_i^t$ for all i. Following (Gilson et al., 2016), the empirical FC consists of two BOLD covariance matrices (see the blue and green matrices in the top box of Fig. S1), without and with time lag:

$$\begin{aligned} \widehat{Q}^0_{ij} &= \frac{1}{T-2} \sum_{1 \leq t \leq T-1} (s_i^t - \bar{s}_i)(s_j^t - \bar{s}_j) \ , \\ \widehat{Q}^1_{ij} &= \frac{1}{T-2} \sum_{1 \leq t \leq T-1} (s_i^t - \bar{s}_i)(s_j^{t+1} - \bar{s}_j) \ . \end{aligned} \tag{1}$$

Pearson correlation can be calculated from the covariances in Eq. (1):

$$K_{ij} = \frac{\widehat{Q}^0_{ij}}{\sqrt{\widehat{Q}^0_{ii}\widehat{Q}^0_{jj}}} \ . \tag{2}$$

C Multivariate Ornstein-Uhlenbeck (MOU) process to model whole-brain dynamics

The network dynamics are determined by two sets of parameters:

Table 1: Table of ROI indices and labels.

Indices	Abbreviation	Name
1/66	right/left CUN	cuneus
2/65	right/left PCAL	pericalcarine gyrus
3/64	right/left LING	lingual gyrus
4/63	right/left LOCC	lateral occipital cortex
5/62	right/left FUS	fusiform gyrus
6/61	right/left SP	superior parietal cortex
7/60	right/left IP	inferior parietal cortex
8/59	right/left SMAR	supramarginal gyrus
9/58	right/left BSTS	bank of superior temporal sulcus
10/57	right/left PCUN	precuneus
11/56	right/left ST	superior temporal cortex
12/55	right/left TT	transverse temporal cortex
13/54	right/left MT	middle temporal cortex
14/53	right/left IT	inferior temporal cortex
15/52	right/left TP	temporal pole
16/51	right/left PREC	precentral cortex
17/50	right/left PSTC	postcentral cortex
18/49	right/left PARC	paracentral lobule
19/48	right/left FP	frontal pole
20/47	right/left CMF	caudal middle frontal cortex
21/46	right/left RMF	rostral middle frontal cortex
22/45	right/left PTRI	pars triangularis
23/44	right/left PORB	pars orbitalis
24/43	right/left POPE	pars opercularis
25/42	right/left SF	superior frontal cortex
26/41	right/left LOF	lateral orbitofrontal cortex
27/40	right/left MOF	medial orbitofrontal cortex
28/39	right/left ENT	entorhinal cortex
29/38	right/left PARH	parahippocampal cortex
30/37	right/left CAC	caudal anterior cingulate cortex
31/36	right/left RAC	rostral anterior cingulate cortex
32/35	right/left PC	posterior cingulate cortex
33/34	right/left ISTC	isthmus of cingulate cortex

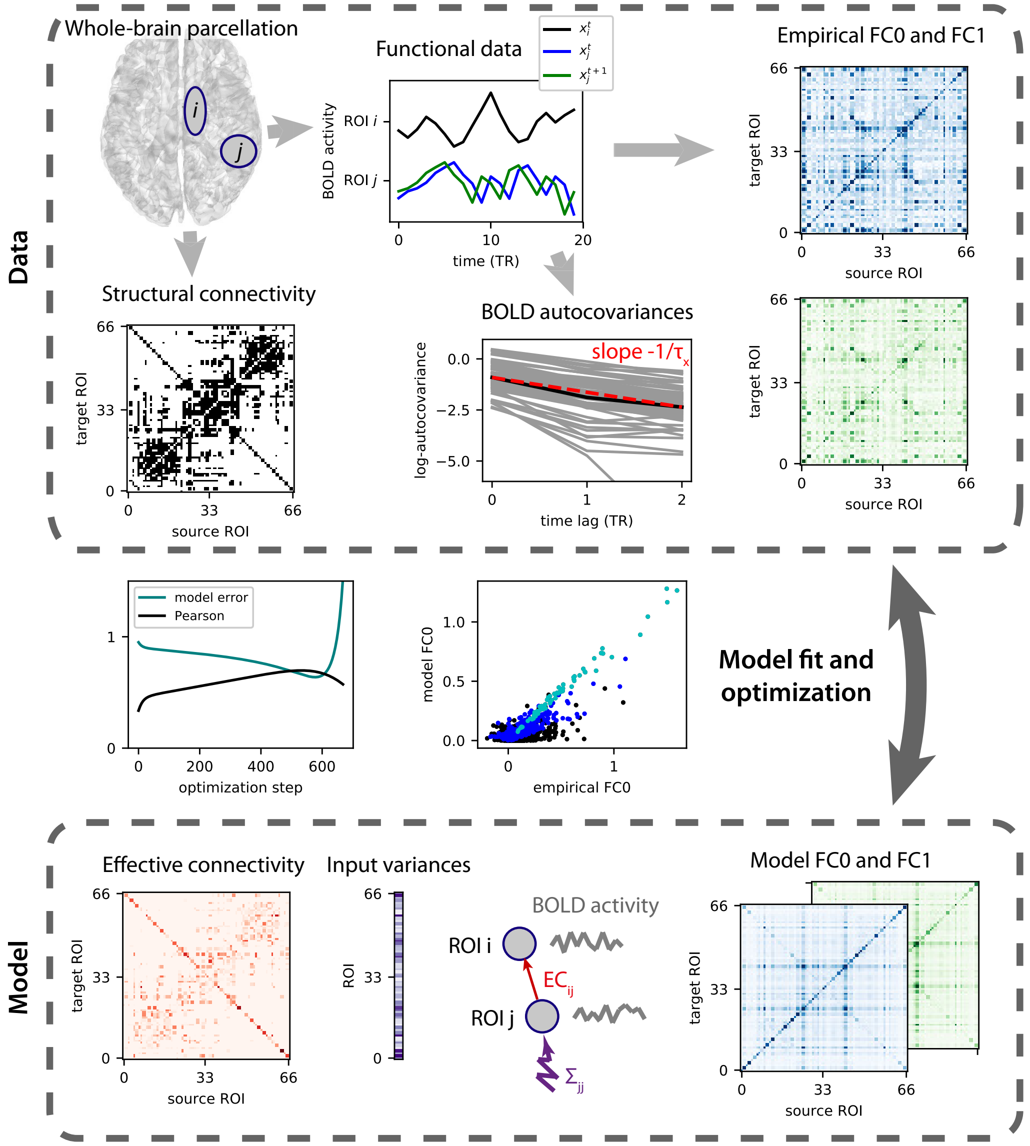

Figure S1: **Dynamic model and optimization procedure to capture BOLD dynamics.** From the BOLD signals of the parcellation (top left in top box), two empirical FC matrices (blue and green matrices) are calculated, both without and with time lag as depicted by the blue and green curve. The dynamic network model describes the activity of the ROIs (only two are represented by the grey circles) and comprises a parameter for the spontaneous activity of each ROI (Σ in the bottom box) and for each directed connection between ROIs (effective connectivity, MOU-EC). The topology of existing connections is determined by the structural connectivity (black matrix in top box). The optimization of the model is similar to a gradient descent during which the model FC matrices are evaluated at each step and compared to their empirical counterparts. From the differences between the corresponding matrices an update of the MOU-EC and Σ parameters is calculated and the procedure is repeated until reaching a minimum for the model error (light gray-blue curves in the left central panel between the boxes), which corresponds to a high value for the Pearson correlation between the matrix elements of the model and empirical FCs (black curve). Before the optimization, the time constant τ_x of the BOLD autocovariances (see log-plot) is evaluated to calibrate the nodal dynamics in the model. Details can be found in (Gilson et al., 2016, 2018).

- the time constant τ_x is an abstraction of the
- the network effective connectivity (MOU-EC) between these ROIs embodied by the matrix C, whose topological skeleton is determined by structural data;
- the local variability embodied in the matrix Σ inputed individually to each of the $N = 66$ ROIs.

The activity variables obey are described by a multivariate Ornstein-Uhlenbeck process. Each activity variable x_i of node i decays exponentially according to the time constant τ_x and evolves depending on the activity of other populations:

$$\mathrm{d}x_i = \big(\frac{-x_i}{\tau_x} + \sum_{j \neq i} C_{ji} x_j\big)\mathrm{d}t + \mathrm{d}B_i \ . \tag{3}$$

Compared to our previous work (Gilson et al., 2016, 2018), we use a different convention for the weights C_{ji} from ROI j to ROI i, in line with graph theory. In matrix notation it now reads

$$\mathrm{d}x = xJ\mathrm{d}t + \mathrm{d}B \ . \tag{4}$$

Here the fluctuating inputs $\mathrm{d}B_i$ are independent and correspond to a diagonal covariance matrix Σ, as represented in purple by the vector of variances Σ_{ii} in the bottom box of Fig. S1. In the model, all variables x_i have zero mean. Their spatiotemporal covariances are denoted by Q^1_{ij}, where $\tau \in \{0, 1\}$ is the time lag, and correspond to the the blue and green matrices in the bottom box of Fig. S1. They can be calculated by solving the consistency equations:

$$J^\dagger Q^0 + Q^0 J = -\Sigma \ , \tag{5}$$
$$Q^1 = Q^0 e^J \ . \tag{6}$$

The first equation is the continuous Lyapunov equation, which can be solved using the Bartels-Stewart algorithm in scipy (??, sci). Here J is the Jacobian of the dynamical system and depends on the time constant τ_x and the network MOU-EC: $J_{ji} = \frac{-\delta_{ii}}{\tau_x} + C_{ji}$, where δ_{ji} is the Kronecker delta and the superscript $\dagger$ denotes the matrix transpose. In the second equation e denotes the matrix exponential.

Ideally, the model should be extended to incorporate subcortical areas and the relevance of input cross-correlations inputs should be evaluated for all ROI pairs (Gilson et al., 2018).

D Parameter estimation procedures

D.1 Lyapunov optimization or natural gradient descent

We tune the model such that its covariance matrices Q^0 and Q^1 reproduce the empirical FC0 and FC1 matrices in Fig. ??B, denoted here by $\widehat{Q}^0$ and $\widehat{Q}^1$, respectively. The uniqueness of this

maximum-likelihood estimation follows from the bijective mapping from the model parameters τ_x, C and Σ to the FC pair (Q^0, Q^1). The iterative optimization procedure for C is similar to the original version (Gilson et al., 2016), which can be related to the concept of "natural" gradient descent (Amari, 1998) that takes the non-linearity of the mapping between J and the matrix pair Q^0 and Q^1 in the second line of Eq. (5). Note that the parameters τ_x and Σ also follow a gradient descent now.

For each session (subject and condition), we calculate the time constant τ_{ac} associated with the exponential decay of the autocovariance averaged over all ROIs using time lags 0 and 1 TR:

$$\tau_{\text{ac}} = -\frac{N}{\sum_{1 \leq i \leq N} a(v_i|u)} = \frac{N\tau_{\text{TR}}}{\sum_i \log(\widehat{Q}^0_{ii}) - \sum_i \log(\widehat{Q}^1_{ii})} \,, \tag{7}$$

where $a(v_i|u)$ is the slope of the linear regression of $v_i = [\log(\widehat{Q}^0_{ii}), \log(\widehat{Q}^1_{ii})]$ by $u = [0, 1]$, and τ_{TR} is the value of the TR in seconds. Note that one can also use more time lags —e.g. up to 2 TRs— to assess the stability of the estimated τ_{ac}, as was previously analyzed for resting-state data (Gilson et al., 2016). The model is initialized with $\tau_x = \tau_{\text{ac}}$ and no connectivity $C = 0$, as well as unit variances without covariances ($\Sigma_{ij} = \delta_{ij}$). At each step, the Jacobian J is straightforwardly calculated from the current values of τ_x and C. Using the current Σ, the model FC matrices Q^0 and Q^1 are then computed from the consistency equations, using the Bartels-Stewart algorithm to solve the Lyapunov equation.

The difference matrices $\Delta Q^0 = \widehat{Q}^0 - Q^0$ and $\Delta Q^1 = \widehat{Q}^1 - Q^1$ determine the model error

$$E = \frac{1}{2}\frac{||\Delta Q^0||}{||Q^0||} + \frac{1}{2}\frac{||\Delta Q^1||}{||Q^1||} = \frac{1}{2}\frac{\sum_{i,j}(\Delta Q^0_{ij})^2}{\sum_{i,j}(\widehat{Q}^0_{ij})^2} + \frac{1}{2}\frac{\sum_{i,j}(\Delta Q^1_{ij})^2}{\sum_{i,j}(\widehat{Q}^1_{ij})^2} \,, \tag{8}$$

where each term - for FC0 and FC1 - is the matrix distance between the model and data covariances, normalized by the latter.

The following Jacobian update can be applied to decrease the model error E at each optimization step similar to a gradient descent:

$$\Delta J = (Q^0)^{-1}[-\Delta Q^0 + \Delta Q^1 e^J] \,, \tag{9}$$

It turns out that a modified update is more robust to empirical noise in practice

$$\Delta J = (Q^0)^{-1}\Delta Q^0 + \Delta Q^0 (Q^0)^{-1} + (Q^1)^{-1}\Delta Q^1 + \Delta Q^1 (Q^1)^{-1} \,, \tag{10}$$

which would corresponds to a proxy error based on the matrix logarithm:

$$\tilde{E} = ||\log(Q^0) - \log(\widehat{Q}^0)|| + ||\log(Q^1) - \log(\widehat{Q}^1)|| \,. \tag{11}$$

See (Insabato et al., 2018) for a comparison of optimization methods. From the Jacobian update ΔJ, we obtain the connectivity update:

$$\Delta C_{ij} = \eta_C \Delta J_{ij} \tag{12}$$

for existing connections only; other weights are forced at 0. We also impose non-negativity for the MOU-EC values during the optimization.

To take properly the effect of cross-correlated inputs into account, the Σ update has been adjusted from the heuristic update in the first study (Gilson et al., 2016) to a gradient descent (Gilson et al., 2018):

$$\Delta\Sigma = -\eta_\Sigma (J^\dagger \Delta Q^0 + \Delta Q^0 J) \ . \tag{13}$$

As with weights for non-existing connections, only diagonal elements of Σ —and possible cross-correlated inputs (Gilson et al., 2018)— are tuned; others are kept equal to 0 at all times.

Last, to compensate for the increase of recurrent feedback due to the updated C, one can also tune the model time constant τ_x as

$$\Delta\tau_x = \eta_\tau \left(\tau_{\mathrm{ac}} + \frac{1}{\lambda_{\max}} \right) \ , \tag{14}$$

where $\lambda_{\max}$ is the maximum negative real part of the eigenvalues of the Jacobian J. The rationale is to avoid an explosion of the network dynamics (when $\lambda_{\max} \to 0$) while letting the model connectivity C develop a non-trivial structure to reproduce FC.

Repeating the parameter updates, the best fit corresponds to the minimum of the model error E.

D.2 Heuristic optimization

Instead of the derivation of the Jacobian in Eq. (10) that takes into account the nonlinearity of the mappings in Eq. (5), one can use a greedy algorithm to optimize the weights to fit an objective measure on the data. Here we update the weights for the model to reproduce the correlation matrix K in Eq. (2):

$$\Delta C_{ij} = \eta_C \Delta K^0_{ij} \ . \tag{15}$$

Note that only correlations ΔK^0_{ij} for existing connections $i \to j$ and $j \to i$ are taken into account in this update.

E Dynamic communicability and flow for network analysis

Following our previous study (Gilson et al., 2018), we firstly define *dynamic communicability* to characterize the network interactions due to the MOU-EC connectivity C, ignoring the input properties Σ. Our definition is adapted to study complex networks associated with realistic (stable) dynamics where time has a natural and concrete meaning. In comparison, a previous version of communicability for graphs (Estrada and Hatano, 2008) relied on abstract dynamics. The basis of our framework is the network response over time, or Green function, which is the basis of the concept of dynamic communicability, which focuses on the temporal evolution of such interactions. Although we focus on the MOU process here, our framework can be easily adapted to distinct local dynamics for which the Green function is known. In addition to the MOU-EC matrix C, the

MOU dynamics is determined by the input properties, so we use the *dynamic flow* in the main text (Figs. 6 and 8).

E.1 Definitions

Formally, dynamic communicability is the "deformation" of the Green function e^{Jt} of the MOU process due to the presence of the (weighted and directed) matrix C, as compared to the Green function $e^{J^0 t}$ corresponding to the Jacobian with leakage only and no connectivity, $J^0_{ij} = -\delta_{ij}/\tau$. It is defined as the family of time-dependent matrices depicted in Fig. 6A:

$$\mathcal{C}(t) = ||J^0||\big(e^{Jt} - e^{J^0 t}\big) \ . \tag{16}$$

The scaling factor $||J^0||^{-1} = ||\int_{t\geq 0} e^{J^0 t} \mathrm{d}t||$ where $||\cdot||$ is the L1-norm for matrices (i.e., sum of elements in absolute value) is used for normalization purpose (Gilson et al., 2018). Recall that $t \geq 0$ here is the time for the propagation of activity in the network, referred to as 'impulse-response time' in the figures.

To incorporate the effect of local spontaneous activity or excitability (inputs in the model), we define the dynamic flow that fully characterizes the complex network dynamics (Gilson et al., 2018). The input statistics of interest for a stable MOU process correspond to the input (co)variance matrix Σ, which are independent parameters from the MOU-EC matrix C. This is represented by the purple arrow in the left diagram of Fig. 6A, indicating that the fluctuation amplitude is individual for each ROI. The Σ matrix may be non-diagonal when ROIs experience cross-correlated noise (Gilson et al., 2018), as represented by the purple dashed arrows. The dynamic flow describes the propagation of local fluctuating activity over time via the recurrent connectivity and is defined by the

$$\mathcal{F}(t) = \sqrt{\Sigma}\, \mathcal{C}(t) \, , \tag{17}$$

where $\sqrt{\Sigma}$ is the real symmetric "square-root" matrix of the input covariance matrix, satisfying $\Sigma = \sqrt{\Sigma}\sqrt{\Sigma}^\dagger$. Dynamic communicability is thus a particular case of the flow for homogeneous input statistics.

From the time-dependent matrices $\mathcal{F}(t)$, we define the total flow that sums all interactions

$$\mathcal{S}^{\mathcal{F}}(t) = \sum_{\{i,j\}} \mathcal{F}_{ij}(t) \ . \tag{18}$$

Total communicability for graphs has been used to define a version of centrality (Benzi and Klymko, 2013). Here the proximity between ROIs correspond to how much flow they exchange. We also define the diversity (akin to heterogeneity) among the ROI interactions in the time-dependent matrices $\mathcal{F}(t)$, which can be seen as a proxy for their homogenization over time:

$$\mathcal{D}^{\mathcal{F}}(t) = \frac{\sigma_{\{i,j\}}[\mathcal{F}_{ij}(t)]}{\mu_{\{i,j\}}[\mathcal{F}_{ij}(t)]} \ , \tag{19}$$

defined as a coefficient of variation where $\mu_{\{i,j\}}$ and $\sigma_{\{i,j\}}$ are the mean and standard deviation over the matrix elements indexed by (i,j).

We also define the input and output flows for each node:

$$
\begin{aligned}
\mathcal{F}^{\text{in}}_j(t) &= \sum_i \mathcal{F}_{ij}(t)\,, \\
\mathcal{F}^{\text{in}}_i(t) &= \sum_j \mathcal{F}_{ij}(t)\,.
\end{aligned}
\tag{20}
$$

E.2 Community detection

To detect communities from $\mathcal{F}(t)$ in Fig. 8 in the main text, we rely on Newman's greedy algorithm for modularity (Newman, 2006) that was originally designed for weight-based communities in a graph. Adapting it here to the flow matrix $\mathcal{F}(t)$ at a given time t, we seek for communities where ROIs have strong bidirectional flow interactions. In the same manner as with weighted modularity, we calculate a null model for MOU-EC:

$$
C^{\text{null}} = \frac{\mathbf{c}^{\text{in}} \mathbf{c}^{\text{out}\dagger}}{\mathcal{S}^C}\;. \tag{21}
$$

Note that we preserve the empty diagonal. The resulting matrix contains from the expected weight for each connection, given the observed input strengths $\mathbf{c}^{\text{in}}$ and output strengths $\mathbf{c}^{\text{out}}$; $\mathcal{S}^C$ is the total sum of the weights in C. Then we caclulate $\mathcal{F}^{\text{null}}(t)$ using Eq. (17) with C^{null} instead of C. Starting from a partition where each ROI is a singleton community, the algorithm iteratively aggregates ROIs to form a partition of K communities denoted by S_k that maximizes the following quality function:

$$
\Phi = \sum_{1 \le k \le K} \sum_{i,j \in S_k} \big(\mathcal{F}(t) - \mathcal{F}^{\text{null}}(t)\big)_{ij} + \big(\mathcal{F}(t) - \mathcal{F}^{\text{null}}(t)\big)_{ji}\,. \tag{22}
$$

At each step of the greedy algorithm, the merging of two of the current communities that maximizes the increase of Φ is performed. Note that communicability-based communities can be defined similarly using $\mathcal{C}(t)$ and the corresponding null model $\mathcal{C}^{\text{null}}(t)$.

References

http://scipy.org/, numpy-scipy python library.

Abraham, A., F. Pedregosa, M. Eickenberg, P. Gervais, A. Mueller, J. Kossaifi, A. Gramfort, B. Thirion, and G. Varoquaux (2014). Machine learning for neuroimaging with scikit-learn. *Front Neuroinform 8*, 14.

Amari, S.-I. (1998). Natural gradient works efficiently in learning. *Neural Computation 10*, 251–276.

Benzi, M. and C. Klymko (2013). Total communicability as a centrality measure. *Journal of Complex Networks 1*, 124–149.

Estrada, E. and N. Hatano (2008). Communicability in complex networks. *Phys Rev E Stat Nonlin Soft Matter Phys 77*, 036111.

Gilson, M., G. Deco, K. Friston, P. Hagmann, D. Mantini, V. Betti, G. L. Romani, and M. Corbetta (2018). Effective connectivity inferred from fmri transition dynamics during movie viewing points to a balanced reconfiguration of cortical interactions. *Neuroimage 180*, 534–546.

Gilson, M., N. E. Kouvaris, G. Deco, and G. Zamora-López (2018). Framework based on communicability and flow to analyze complex network dynamics. *Phys Rev E 97*, 052301.

Gilson, M., R. Moreno-Bote, A. Ponce-Alvarez, P. Ritter, and G. Deco (2016). Estimation of directed effective connectivity from fMRI functional connectivity hints at asymmetries of cortical connectome. *PLoS Comput Biol 12*, e1004762.

Hagmann, P., L. Cammoun, X. Gigandet, R. Meuli, C. J. Honey, V. J. Wedeen, and O. Sporns (2008). Mapping the structural core of human cerebral cortex. *PLoS Biol 6*, e159.

Hlinka, J., M. Palus, M. Vejmelka, D. Mantini, and M. Corbetta (2011). Functional connectivity in resting-state fMRI: is linear correlation sufficient? *Neuroimage 54*, 2218–2225.

Insabato, A., J. P. Cunningham, and M. Gilson (2018). Bayesian estimation for large scale multivariate ornstein-uhlenbeck model of brain connectivity. *arxiv*, 1805.10050.

Mantini, D., U. Hasson, V. Betti, M. G. Perrucci, G. L. Romani, M. Corbetta, G. A. Orban, and W. Vanduffel (2012). Interspecies activity correlations reveal functional correspondence between monkey and human brain areas. *Nat Methods 9*, 277–282.

Newman, M. E. J. (2006). Modularity and community structure in networks. *Proc Natl Acad Sci U S A 103*, 8577–8582.
